# Molecular basis of N_2_ fixation in a hyperthermophilic archaeon

**DOI:** 10.1038/s41467-026-77173-0

**Published:** 2026-09-08

**Authors:** Nevena Maslać, Mustafa Rasim Törer, Pauline Bolte, Tristan Wagner

**Affiliations:** 1 Max Planck Institute for Marine Microbiology, Celsiusstrasse 1, Bremen, Germany; 2 Univ. Grenoble Alpes, CEA, CNRS, Institut de Biologie Structurale, Grenoble, France; 3 Present Address: Department of Functional and Evolutionary Ecology, Archaea Biology and Ecogenomics Unit, University of Vienna, Djerassiplatz 1, Vienna, Austria; 4 Present Address: Evolutionary Biochemistry Group, Department of Biology, Marburg University, Karl-von-Frisch-Strasse 8, Marburg, Germany

**Keywords:** Biocatalysis, Archaea, X-ray crystallography, Enzymes

## Abstract

Exploring the natural diversity of phylogenetically distant nitrogenases is crucial for gaining new insights into the mechanism of atmospheric N_2_ fixation and unlocking biotechnological developments in sustainable ammonia production. Here, we investigated the N_2_-fixing system of *Methanocaldococcus infernus*, a deep-sea hyperthermophilic archaeon growing diazotrophically at 92 °C. This natively isolated nitrogenase has a melting temperature close to the water boiling point, with an extrapolated specific activity superior to mesophilic homologues. The crystal structures obtained at near-atomic resolution present the most simplified known nitrogenase, harbouring strategic hot spots for thermostability. It combines the structural traits of all three known nitrogenase isoforms, reinforcing the proposal that ancestral nitrogenases were more similar to the archaeal enzyme than to the bacterial homologues. In contrast to structural homologues, the electron-transferring metallocofactor “P-cluster” is trapped in a rare state awaiting electron delivery, providing a detailed picture of the physiological state. The active site, harbouring the FeMo-cofactor catalyst, exhibits a mixture of the resting and “turnover” states, previously described solely in the bacterial vanadium and iron-only nitrogenases. Therefore, these results unify a mechanistic principle of all nitrogenases and highlight the advantages of the hyperthermostable nature of the archaeal enzyme, opening new avenues for further understanding of how nature splits the N_2_ triple bond.

## Abstract (web summary)

Nitrogenases are enzymes that convert atmospheric N_2_ into ammonia, thus providing bioavailable nitrogen to living organisms. Here, Maslać et al. describe a highly thermostable, simple nitrogenase from a deep-sea microbe, revealing common mechanistic principles in these enzymes.

## Introduction

Performed exclusively by diazotrophic microbes, biological N_2_-fixation provides half of the nitrogen to the living organisms of our planet^1–3^. Alongside a minor contribution from geochemical discharge through lightning, diazotrophy is the only known natural reaction capable of breaking the triple bond of atmospheric N_2_ to generate the bioavailable fertiliser and biofuel ammonia (NH_3_) under ambient pressure and temperature^4^. In comparison, the industrial Haber-Bosch process requires extreme conditions (200 bar and 400–500 °C) and generates 1–2% of the global CO_2_ emissions^5–8^. Greener alternatives for decarbonising NH_3_ production could be inspired by biological systems, for instance, to transform eukaryotes into diazotrophs^9,10^. For this, a deep understanding of the biological N_2_ fixation mechanism, which remains one of the most complex unanswered questions in bioinorganic catalysis, is necessary and must be addressed.

**Fig. 1:**
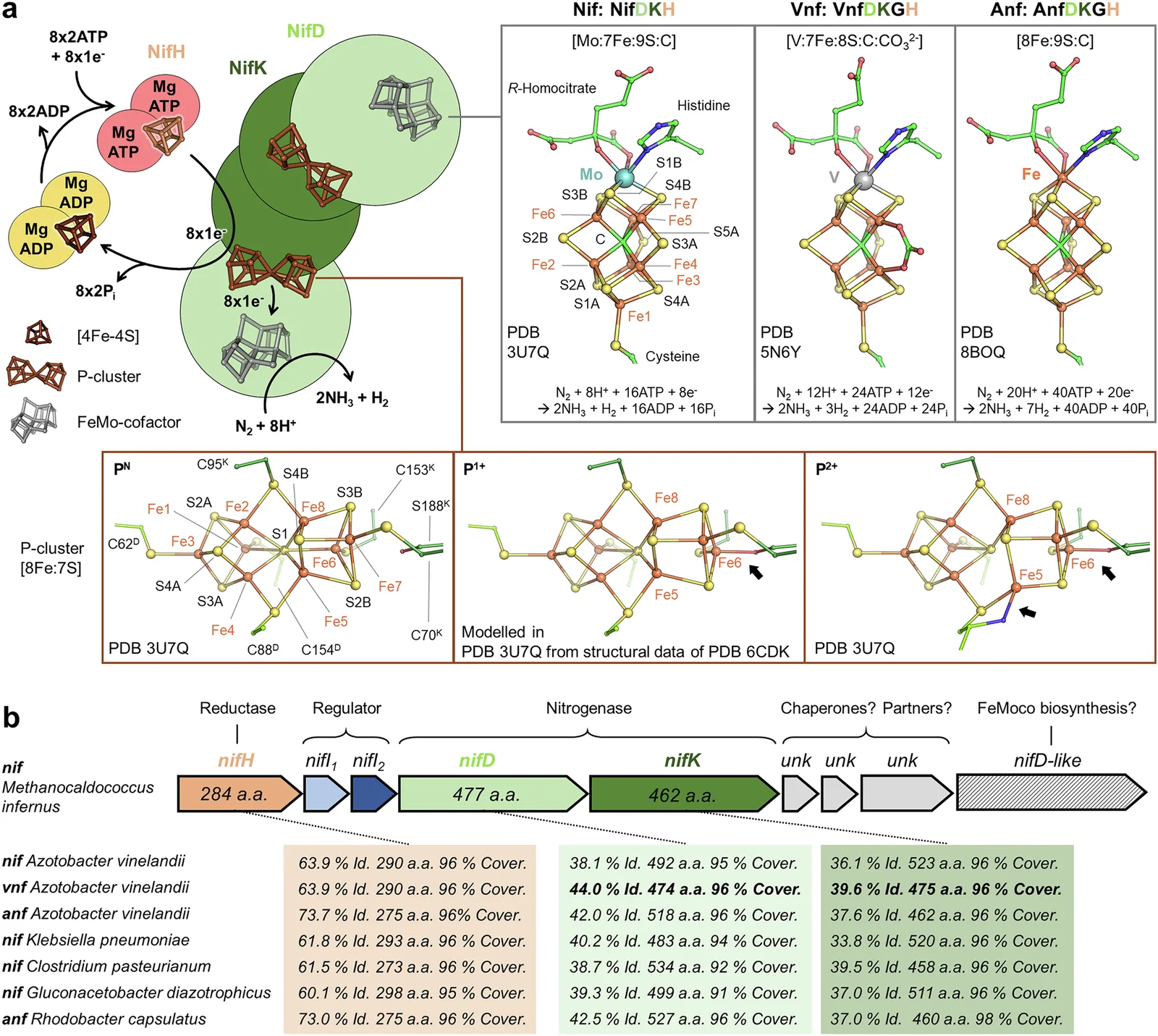
Overview of the nitrogenase cycle and sequence comparison between structural homologues and *M. infernus nif* system. **a** Presentation of the nitrogenase catalytic cycle in which reduced NifH loaded with MgATP provokes the displacement of an electron from the P-cluster to the FeMoco following the electron-deficient mechanism previously proposed^17^. The P-cluster in its P^1+^ state switches back to its fully reduced P^N^ state by uptaking the electron from NifH upon ATP hydrolysis. Phosphate release (P_i_) has been shown to be the rate-limiting step of the cycle^97^. Different types of catalysts exist exhibiting different chemical properties^98^ (right panel): the FeMoco, FeVco, and FeFeco carried by NifDK, VnfDK, and AnfDK, respectively. P-cluster states are displayed in the bottom panel. The overall reaction model comes from ref. ^99^. **b** *nif* operon in *M. infernus* containing three putative genes of unknown function (*unk*^38^,). Amino acid lengths (a.a.) are indicated for the protein encoded by *nifDKH*, and their protein sequence identity (Id.) /coverage (Cover) is compared to homologues. Only methanogens and anaerobic methanotrophs are diazotrophs^100^ in Archaea. Therefore, they are not only key actors in the carbon cycle, generating or degrading approximately 1 Gt of methane annually^101^, but also contribute to the nitrogen cycle in specific niches^31^. *Methanococcales* depend exclusively on H_2_ and CO_2_ (or formate) to generate methane, a catabolism that has been described as at the thermodynamic limits of Life^102^. Despite these low energy yields, they can sustain the energetic toll of at least 16 ATP per fixed N_2_ thanks to the post-translational regulation system^36^ and reallocation of cellular resources^37^. *nifB*, critical for the FeMoco synthesis, is at a different position in the genome.

The sole enzymatic system capable of fixing N_2_ is the ATP-dependent nitrogenase, composed of NifH and NifDK. NifH contains a [4Fe-4S]-cluster capable of delivering an electron upon ATP hydrolysis to NifDK^3,11^. Electrons are ultimately transferred to the FeMo-cofactor bound on NifD (FeMoco, [Mo:7Fe:9S:C], one of the most complex enzymatic metallocofactors known in living systems) through the P-cluster ([8Fe:7S]). FeMoco is asymmetric, adopting a trigonal prismatic (*D*3h) geometry^12,13^ with two prism halves linked by three bridging sulfides, containing a central μ₆-carbide^14^ and an apical molybdenum atom. The P-cluster, harboured at the NifDK interface, consists of two symmetric [4Fe:4S] cubanes that share a central μ_6_-sulfide (S) in its reduced state. During catalysis, the P-cluster has been proposed to oscillate between a fully reduced P^N^ and the electron-depleted P^1+^ state (following the accepted deficit-spending mechanism, Fig. 1a)^15–18^. Midpoint potentials for the P^N^/P^1+^ and P^1+^/P^2+^ couples have been reported to be − 310 mV at pH 7.5–8.0^16^. While the P-cluster can be further oxidised to P^2+^ and P^3+^ states^19,20^, there is no evidence that these transitions happen during the catalytic cycle^15,19^. Moreover, the P^3+^ state is considered irreversible^21^. FeMoco can also oscillate between multiple different states during catalytic turnover, including the stable *S* = 3/2 resting state (FeMoco^N^) and the one-electron oxidised state (FeMoco^ox^)^16^. There are two more isoforms of nitrogenase in which FeMoco is replaced by a FeV-cofactor (Vnf system, [V:7Fe:8S:C:CO_3_^2-^]) or a FeFe-cofactor (Anf system, [8Fe:9S:C], Fig. 1a)^22,23^.

Spanning more than 60 years, research integrating structural, spectroscopic, and biochemical data has deepened our understanding of the complexity underlying biological N₂ reduction. Yet, the field remains largely shaped by studies on a narrow set of diazotrophic organisms. Most of the knowledge stems from *Azotobacter vinelandii*, whose disproportionately large set of *nif* genes^24^ may hinder our perception. Phylogenetically distant diazotrophs, on the other hand, with distinct lifestyles and metabolic requirements, often rely on a minimal set of genes for N₂ fixation. Such a shift in perspective not only fortifies ongoing synthetic biology initiatives^9,25^ but also offers the opportunity to clarify the mechanistic principles of N₂ fixation by distinguishing conserved strategies and revealing unanticipated modes of adaptation within nitrogenase catalysis.

In this work, we explore the distant nitrogenase from marine *Methanococcales*, which has been established by phylogenetic studies to resemble the hypothetical progenitor of nitrogenase systems^26–30^. Diazotrophic *Methanoccocales* have been highlighted for their environmental importance as primary N_2_-fixers in some ecological niches^31–33^, successful transplantation of part of their nitrogenase system in plants^34,35^, differences in the regulation of their nitrogen-assimilation node^36–40^, and for their ability to fix N_2_ at the highest temperature recorded so far of 92 °C^41^. By studying the nitrogenase from *Methanocaldococcus infernus*, we found molecular adaptive traits for hyperthermostability and shared structural characteristics with all three known nitrogenase isoforms, providing new insights into the N_2_-fixation mechanisms.

## Results

### Native isolation of a hyperthermostable nitrogenase

**Fig. 2:**
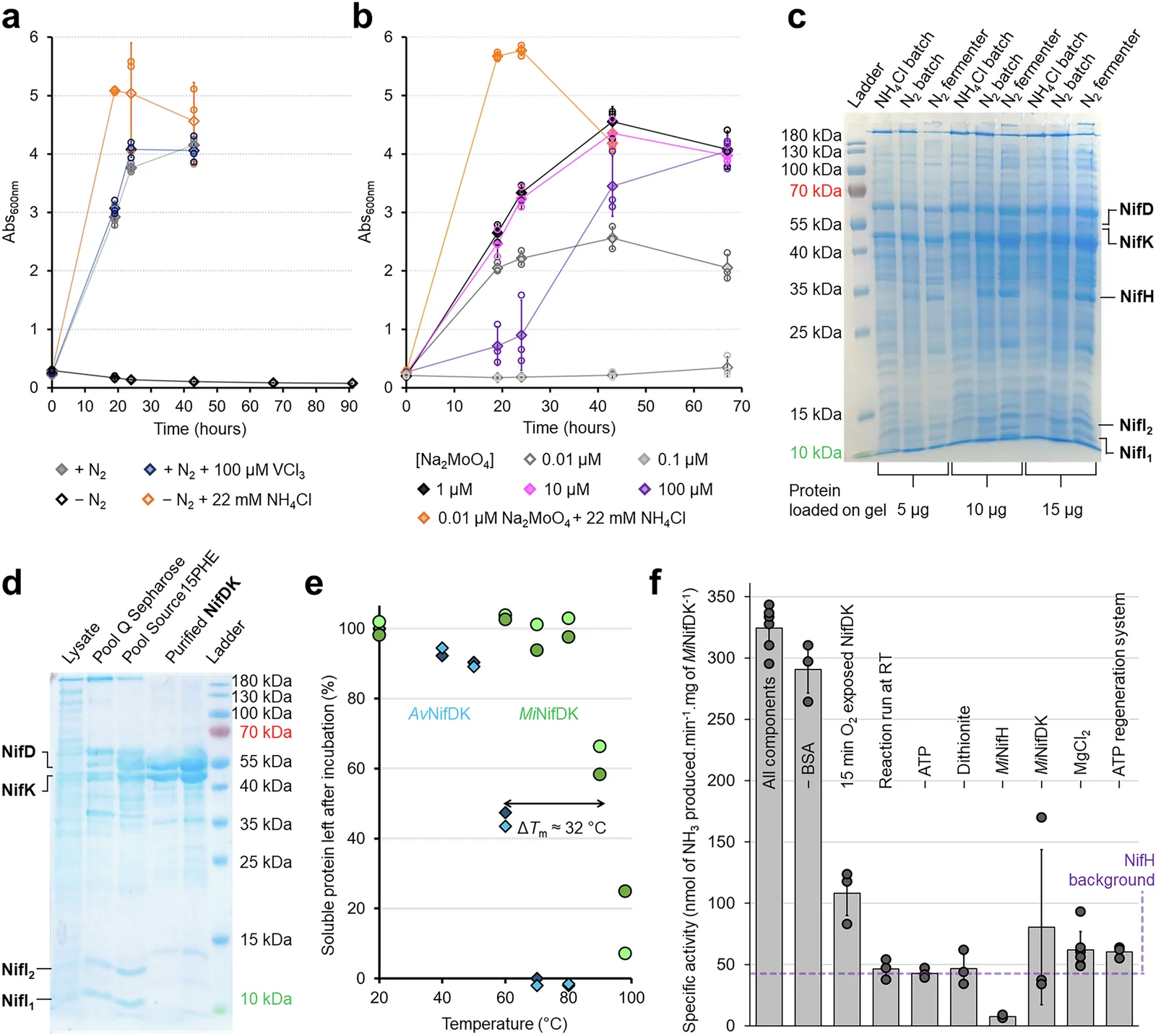
Isolation of an active hyperthermostable nitrogenase from the diazotroph *M. infernus*. **a** Diazotrophic growth *M. infernus* on 10 µM Na_2_MoO_4_ and (**b**) its molybdenum dependency. All tested conditions contain 0.5 bar (0.49 atm) of N_2_ (except for the controls in panel a), 100 µM Na_2_WO_4_ and were grown at 75 °C. Colour coding of the different experiments is indicated below the graphs. **c** *M. infernus* cell extracts grown on NH_4_Cl versus N_2_ in batch culture or in a fermenter showing a natural overexpression of the *nif* system. **d** Native purification of *Mi*NifDK. To separate the regulator *Mi*NifI_1,2_ from NifDK^49^, 2-oxoglutarate and MgATP were added to the Source^TM^ 15PHE pool before further separation by anionic exchange chromatography. 3 µg of proteins were loaded on each well, the NifDK sample close to the ladder being a fraction slightly more contaminated^49^. **e** Thermostability assay of *Av*NifDK (blue) and *Mi*NifDK (green), presenting an estimated *T*_m_ of 60.1 °C and 92.3 °C, respectively. Data from *Av*NifDK come from Maslać et al. 2024^50^. **f** Activity assay of *Mi*NifDKH performed at 50 °C under 100% N_2_ at pH 7.0 for 30 min. Experiments in (**a**, **b**) were performed in triplicate. Experiments for panel (**e**) were performed in duplicate. Experiments for panel (**f**) were performed in triplicate, except for experiments representing all components and without MgCl_2_, which were performed with six replicates. All error bars correspond to the standard deviation.

With its minimal *nif* operon (Fig. 1b), *M. infernus* presents remarkable behaviour during diazotrophic lifestyle with a doubling time of 2 hours under complete autotrophy (Fig. 2a), at a maximum temperature of 91.7 °C, withstanding temperatures as high as 93 °C, though viability is lost after 24 h (Supplementary Fig. 1a). High N_2_ partial pressure (0.49 atm) is required for optimal diazotrophic growth (Fig. 2a and Supplementary Fig. 1b). This behaviour might come from a low solubilisation of N_2_ in the medium at 75 °C, or could underline a competitive inhibitory effect of H_2_ versus N_2_ as reported for *A. vinelandii* nitrogenase^42^.

Diazotrophic growth of *M. infernus* is molybdenum dependent (Fig. 2b), insensitive to VCl_3_ addition (Fig. 2a), and exhibits an outstanding tolerance to tungstate: up to 1000 times higher than what has been observed in *A. vinelandii* or *Methanococcus maripaludis*, a mesophilic *Methanococcales*^43,44^. The latter trait, also reported on the thermophile *Methanothermococcus thermolithotrophicus*^37^, might be an adaptation to the environmental conditions. Despite a closer sequence homology to a VFeco-containing nitrogenase (*Av*VnfDK, Fig. 1b), the molybdenum-dependent diazotrophic growth suggests the utilisation of a FeMoco.

To understand the true nature of *M. infernus* nitrogenase, rather than relying on recombinant systems, we studied the enzyme directly isolated from diazotrophically grown cells expressing naturally high quantities of the N_2_-fixation machinery^45,46^ (Fig. 2c). During the purification, *Mi*NifDK coeluted with *Mi*NifI_1,2_, a regulatory complex belonging to the P_II_-family proteins detecting cellular energy (i.e., ATP/ADP/AMP ratio) and cellular nitrogen availability (i.e., 2-oxoglutarate/glutamate/glutamine ratio)^36,40,47^. *Mi*NifI_1,2_ binding to *Mi*NifDK is most likely due to the decrease in MgATP and 2-oxoglutarate concentration during cell lysis (Fig. 2d)^48^. As expected, the addition of these ligands releases *Mi*NifI_1,2_ inhibition and allows *Mi*NifDK separation and isolation (Supplementary Fig. 1c and Fig. 2d). The identification and structural characterisation of *Mi*NifI_1,2_ alone or in complex with *Mi*NifDK have been recently reported^49^.

The melting temperature (*T*_m_) of *Mi*NifDK was estimated to be 92.3 °C, which is roughly 32 °C higher than that of *Av*NifDK reported previously using the same method^50^ (60.1 °C, Fig. 2e). This is consistent with the diazotrophic growth at 91.7 °C and the high thermostability recorded from the recombinantly produced *Mi*NifH (Supplementary Fig. 1d) with a *T*_m_ of 82.5 °C^50^. Unlike nitrogenases derived from mesophilic organisms (i.e., *A. vinelandii*), NH_3_ production is undetectable when the *M. infernus* whole nitrogenase system is assayed at room temperature (Fig. 2f), reflecting adaptations to contrasting ecological niches and hyperthermophilic adaptation. When the temperature is increased to 50 °C, the maximal temperature allowed by the ATP-regeneration system^51^, a specific activity of 280 ± 18.2 nmol of NH_3_ produced.min^−1^.mg of *Mi*NifDK^−1^ was measured (Fig. 2f). Assuming that the activity follows the Arrhenius law, then this extrapolation would lead to an activity of 2240 nmol of NH_3_ produced.min^−1^.mg of *Mi*NifDK^−1^ at 80 °C, which would be the highest nitrogenase activity ever measured ( ≈ 500–900 nmol of NH_3_ produced.min^−1^.mg^−1^ for native *Av*NifDK^52–54^). The temperature-dependent activity suggests that the enthalpic stabilisation that keeps the structure rigid also prevents the local flexibility needed for substrate turnover, stalling the enzyme at lower temperatures.

### General structural features

The strong reductant dithionite (S_2_O_4_^2-^, DT) is routinely included in nitrogenase purifications, although recent studies have suggested that dithionite may be a non-innocent reductant for metalloproteins^55^. Thus, here, the relatively mild reductant dithiothreitol was added during anaerobic purification and crystallisation, leading to a partially oxidised preparation (Supplementary Fig. 1e). Two crystalline forms named A (monoclinic) and B (triclinic) were obtained, yielding high-quality crystals, which allowed the refinement of the structures to a resolution of 1.37 Å and 1.21 Å, respectively (Supplementary Table 1).

**Fig. 3:**
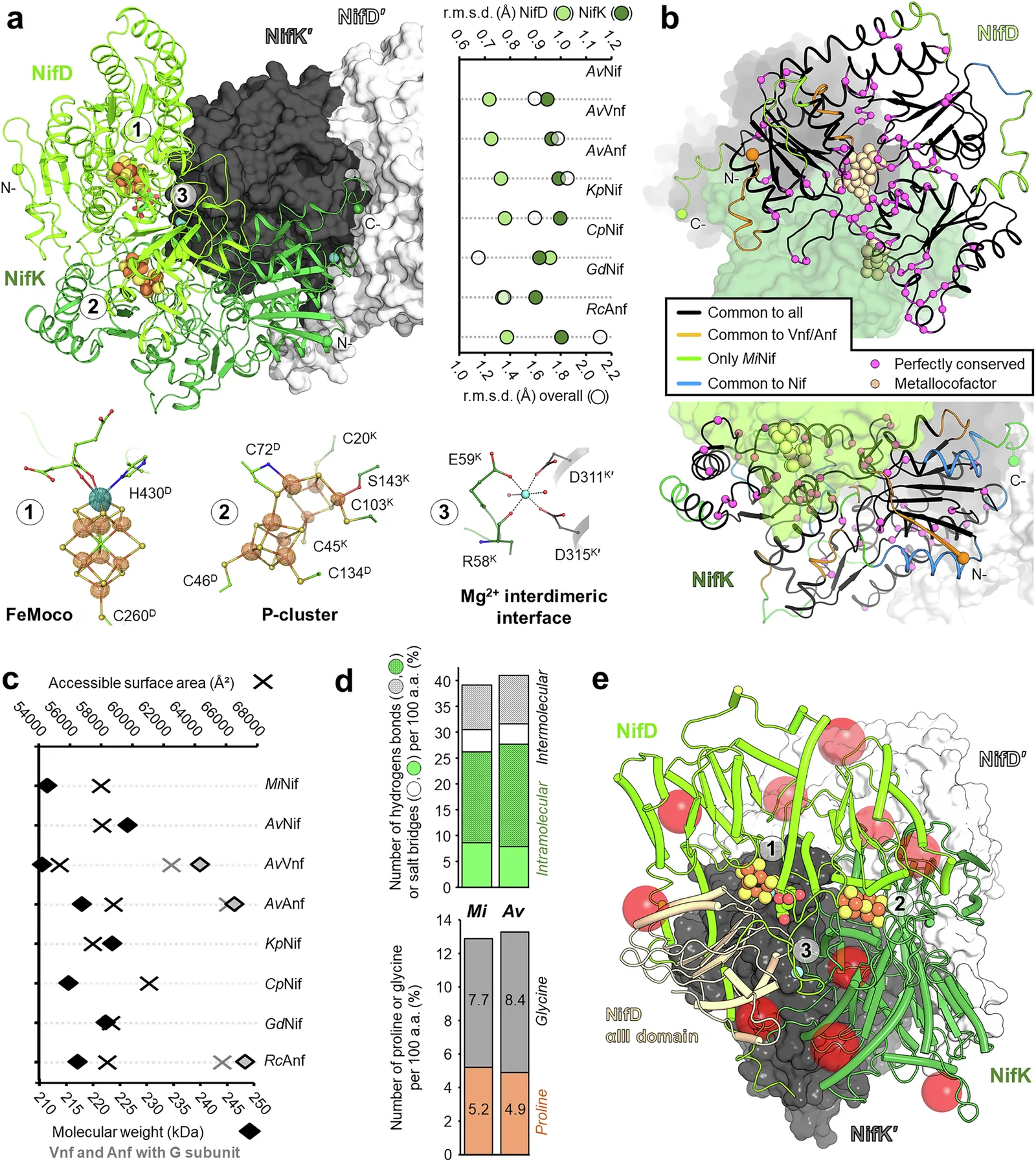
Structural conservation and thermostability features of *Mi*NifDK. **a** *Mi*NifDK overall view with one NifDK in cartoon and the other in surface. Metallocofactors are displayed as balls and sticks with a close-up (bottom panel). Anomalous difference Fourier maps for molybdenum (blue mesh/surface) and iron (orange mesh/surface) are contoured at 6 and 8 σ, respectively and generated by comparing the anomalous signals collected at energies before and at the peak of molybdenum (19.9 keV, Mo K-edge remote and 20.1 keV, Mo K-edge peak) and iron (6.00 keV, Fe K-edge remote and 7.16 keV, Fe K-edge peak fluorescence, seeMethods). Superscript ´ is used to differentiate between the two different subunits of NifK and NifD. The right graph represents the root mean square deviations between *Mi*NifD, *Mi*NifK or *Mi*NifD_2_K_2_ (overall) and respective structural homologues. **b** Secondary structure conservation between *Mi*NifDK and homologues after manual inspection. *Mi*NifD (top) and *Mi*NifK (bottom) are shown as cartoons with the other subunits shown as surface and coloured as in (**a**) with perfectly conserved residues represented as magenta spheres. **c** Comparison of the surface area and molecular weight between the overall structure of *Mi*NifDK and structural homologues. **d** Comparison of the number of hydrogen bonds/salt bridges and the Gly/Pro between *Mi*NifDK and *Av*NifDK. **e** *Mi*NifDK is represented as in panel (**a**) with helices in tubes. Local hot spots are indicated by red transparent spheres and are individually described in Supplementary Fig. 6.

The overall structure presents the canonical NifD_2_K_2_ assembly in which dimerisation occurs via NifK^56^ (Fig. 3a). As observed by sequence identity conservation (Fig. 1b) and phylogenetic analyses^29^, *Mi*NifDK is not structurally more similar to any particular nitrogenase homologue (Figs. 3a, 1b), limiting its assignment to a Nif, Vnf (shorten as VnfDK) or Anf (shortened as AnfDK) system. To resolve this uncertainty and to confirm whether this archaeal enzyme belongs to a Nif system, X-ray diffraction anomalous-based metal identification was conducted. The molybdenum signature was unambiguously confirmed by comparing the anomalous signal from datasets collected at energies before (19.9 keV, Mo K-edge remote) and at the peak of fluorescence of Mo (20.1 keV, Mo K-edge peak, see Methods, Fig. 3a and Supplementary Table 1). A similar experiment was conducted on the Fe K-edge (7.16 keV, Fe K-edge peak and 6.00 keV, Fe K-edge remote, see Methods), providing high confidence in the Fe signature for the FeMoco and the P-cluster (Fig. 3a site numbers 1 and 2, respectively). The absence of signal excluded the presence of an Fe atom at the NifK/NifK′ interface (site number 3 on Fig. 3a), also called the 16^th^ Fe site^54,57^, which adequately fits a Mg^2+^ as modelled in *Av*VnfDK and *Av*AnfDK^52,58^.

*Mi*NifDK is a FeMoco-containing nitrogenase, but it shares structural features with both Vnf and Anf systems (Fig. 3b). Among shared structural traits are the N-terminal parts of *Mi*NifD and *Mi*NifK, some loops and alpha-helices, and short sections, while structural similarity with Nif is rather limited to a loop in NifD and a few sections in NifK. The C-terminal parts and some peripheral loops of NifD and NifK are unique to *M. infernus* (Fig. 3b). The leftover core conserved across all structural homologues (representing 84.6 and 74.0% of the residues in *Mi*NifD and *Mi*NifK, respectively) contains identical residues maintaining structural integrity (glycine and proline), binding the metallocofactor and involved in catalysis. In addition, conserved positions are numerous at dimeric interfaces, probably to maintain important interactions for the quaternary structure.

Deep structural comparison with *Av*NifDK provides a rational basis to explain the hyperthermostability of *Mi*NifDK (Fig. 2e). The overall structure of *Mi*NifDK represents the most simplified scaffold compared to Nif bacterial homologues (Fig. 3c and Supplementary Figs. 2–4). This results in a reduced overall size, comparable with VnfDK without the VnfG subunit, but does not decrease the surface exposed to the solvent (e.g., no reduction in unstable loops or domains). General traits expected to increase overall stability (i.e., additional inter/intramolecular hydrogen bonding network and salt bridges, reduction of glycine and increase of proline, clusters of hydrophobic residues) are only observable to some extent between *Mi*NifDK and *Av*NifDK (Fig. 3c, d and Supplementary Fig. 5). Hence, we propose that the hyperthermostability is the consequence of a rearrangement of salt bridges/hydrogen bonds and hydrophobic clustering at strategic points. These “hot spots” for structural stability would reinforce the local contacts, while the reallocation of proline and glycine would allow the protein to balance rigidity and flexibility where it is needed (Fig. 3e and Supplementary Figs. 5b and 6). Two of these points are located on the αIII domain (composed of residues 2-32 and 334-430 in *Mi*NifD based on ref. ^59^), known for its high intrinsic flexibility and participating in FeMoco delivery and catalysis^60–63^. It is worth noting that the hot spots are mostly located on the surface, distant from the FeMoco and P-cluster sites, suggesting that the active site and electron transfer path are already highly stabilised or depend on intrinsic flexibility for optimal catalysis.

### The electron transfer path

**Fig. 4:**
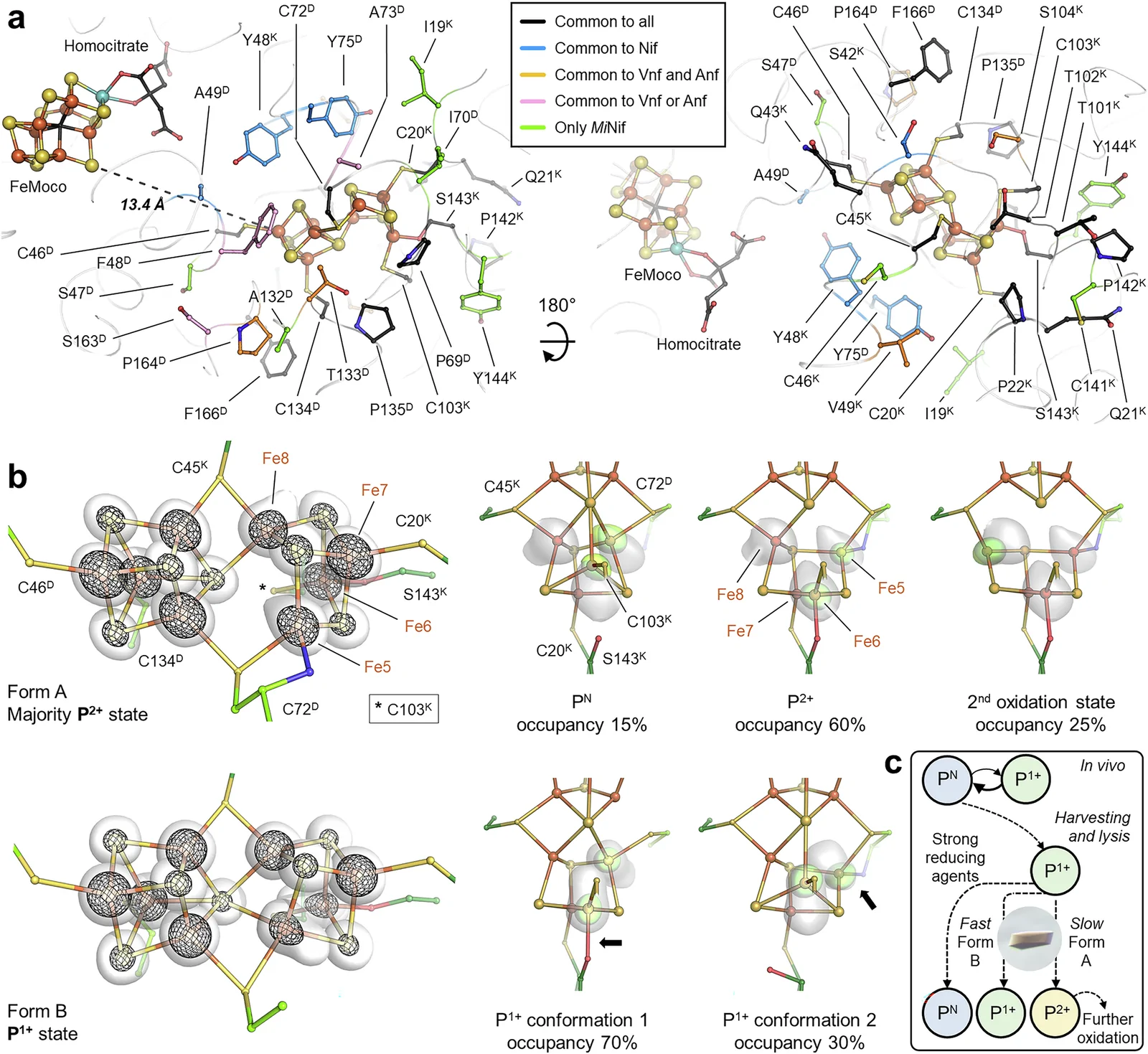
P-cluster site. **a** Conservation of the P-cluster surroundings in *Mi*NifDK and structural homologues. The protein backbone is shown as a cartoon, with residues which are part of the primary and secondary coordination spheres displayed as sticks. Metallocofactors are displayed as spheres and sticks. A colour coding of carbon shows the conservation among *Av*NifDK, *Av*VnfDK and *Av*AnfDK. The shortest distance allowing electron transfer between both metalloclusters is shown by a dashed line. **b** Close-up of the P-cluster in the form A (top) and form B (bottom) with Fe and S from the metallocofactor displayed as spheres and residues binding the P-cluster as sticks. In the left panel, 2*F*_o_−*F*_c_ maps are overlaid as a transparent surface (3.5-σ) and black mesh (8-σ). Right panels illustrate the interpretation of the mixture of different states deconvoluted as a P^N^, P^2+^ and a 2^nd^ oxidation state of the P-cluster for the form A (top) and two alternative conformations of the P^1+^ state for the form B (bottom). The 2*F*_o_−*F*_c_ contoured to 2 σ is presented as a transparent white surface. An omit map was generated for Fe5, Fe6 or Fe8 for the corresponding P-cluster, and the resulting *F*_o_−*F*_c_ maps are presented as a green transparent surface contoured to 7-σ, 30-σ, 10-σ, 32-σ, and 11-σ for P^N^, P^2+^, 2^nd^ oxidation state, P^1+^ alternative conformer A, and P^1+^ alternative conformer B, respectively. Arrows point to the difference in the bond between the two P^1+^ conformations. **c** Scheme illustrating the proposed scenario for how the different isolated states of the P-cluster were obtained. Here, due to the absence of a reducing agent and fast crystallisation, the crystalline form B kept the P^1+^ state, while the crystalline form A has probably been progressively oxidised to P^2+^ and the 2^nd^ oxidation state because of crystallisation delays.

A comparison of the electron transfer path within *Mi*NifDK and the structural homologues from *A. vinelandii* shows a few divergences in the vicinity of the P-cluster, with some positions in the outer coordination shell shared with *Av*VnfDK/*Av*AnfDK and not *Av*NifDK (Fig. 4a). Most importantly, the relay involved in the electron transfer from the P-cluster to the FeMoco is identical to that of the Nif system instead of Vnf/Anf (i.e., the Tyr48^K^/Ala49^D^ pair in *Mi*NifDK instead of the Phe59^K^/Cys52^D^ pair in *Av*Vnf and Phe48^K^/Cys52^D^ in *Av*Anf; with superscripts ^K^ and ^D^ indicating residues belonging to NifK/VnfK/AnfK and NifD/VnfD/AnfD respectively)^52,58^. As expected, the P-cluster-anchoring cysteines and a few residues pointing towards the NifH binding surface are conserved.

We inspected the redox state(s) of the P-cluster by analysing its coordination. The form A mainly exhibits the P^2+^ state modelled with an occupancy of 60%. This oxidised state is recognisable by the Fe6 and Fe5, respectively, bound to the hydroxyl-Ser143^K^ and backbone amide of Cys72^D^ main chain, and a bond loss with the central sulfide (Fig. 4b and Supplementary Fig. 7a)^16^. The remaining 40% are composed of a mixture of two other states. The second conformer is typical of a P^N^ state, with electron densities on Fe6 and Fe5 disengaged from the protein ligands, bound to the central sulfide. As-isolated *Av*NifDK purified in the absence of reducing agents has been reported to only contain the P^2+^ state^64^, however, a similar mixture of P^N^ and P^2+^ was seen in other systems^14,65^. The third conformer comes from an electron density deviation around Fe8, causing bond breakage with the central sulfide (referred to as the second oxidised state, Fig. 4b and Supplementary Figs. 7, 8). The same displacement was observed in the P^2+^ state of the nitrogenase from *Gluconacetobacter diazotrophicus* (*Gd*NifDK), where Fe8 is coordinated by Tyr98^K^^66^, being replaced by Phe99^K^ in *Av*NifDK and Val49^K^ in *Mi*NifDK (Supplementary Fig. 7b). This displacement is assumed to counterbalance the loss of the Fe6-Ser coordination due to serine substitution to alanine in *Gd*NifDK, and was proven by exchanging the pair Phe99^K^/Ser188^K^ to Tyr99^K^/Ala188^K^ in *Av*NifDK^67^. In *Mi*NifDK, Fe8 displacement is stabilised by a water molecule placed at the same position as the hydroxy group of the Tyr98^K^ in *Gd*NifDK (Supplementary Figs. 7b, 8). Based on the electron density profile and structure refinement, we propose that this state could imply a further oxidation of the P-cluster in a P^3+^, and we suspect its preservation to be due to the high enzyme stability caging the oxidised cluster and preventing its collapse. This hypothesis could be further tested using spectroscopic approaches (e.g., electron paramagnetic resonance experiments), which are beyond the scope of the present study.

In contrast to form A, which mostly contains the P^2+^ state, form B contains the P^1+^ state (70% occupancy of conformation 1) in which Fe6 is bound to Ser143^K^ and Fe5 is bound to the central sulfide (Fig. 4b and Supplementary Figs. 7a, 8). It is a very rare state, difficult to trap without redox-mediating agents^19,68^. Elongated electron densities emanating from Fe6 and Fe5 suggest an alternative conformation of the P^1+^ state with an estimated occupancy of 30% (conformation 2). In this conformer, Fe5 is now bound to the amide backbone of Cys72^D^ and Fe6 is bound to the central sulfide. Residues in the surroundings of the P-cluster, including the electron relay, are superposable between both forms (Supplementary Fig. 9), suggesting that the P^1+^ state does not promote drastic conformational changes that would impact the FeMoco centre, such as internal cross-communication.

The different observed redox states between form A and B are rationalised as follows: (*i*) The crystalline forms might have selected a *Mi*NifDK population trapped in a particular state. An explanation challenged by the fact that both structures are superimposable (overall root mean square deviation of 0.226 Å for 1,723 aligned Cα). (*ii*) Form A comes from a low-concentrated sample with crystals appearing after a month. In contrast, form B was obtained from a high-concentrated sample, and crystals were immediately flash frozen after appearing within a few days. (Fig. 4c). This explanation implies that during harvesting and lysis, the extracted *Mi*NifDK harboured in the majority a P^1+^ state, which was stable enough to be maintained over the purification and fast crystallisation by the intrinsic enzyme rigidity and the mild reductant dithiothreitol. Dithiothreitol did not cause the P-cluster reduction to P^N^ in contrast to strong reducing agents such as dithionite (Supplementary Fig. 1e)^16^. The crystallisation delay in form A would have provoked a further uncontrolled oxidation event (e.g., partial O_2_ contamination in the glove box), leading to the appearance of the P^2+^ and the 2^nd^ oxidised state. The very low 15% occupancy of the P^N^ in form A might be due to X-ray radiation or a mixture of P^1+^ states. Altogether, the crystal structures show the overall spectrum of states of the P-cluster critical to understanding the enzyme mechanism, and further studies will document this landscape of redox states with implications for the FeMoco site.

### A FeMoco site captured in resting and “turnover” states underscores common catalytic principles of nitrogenases

**Fig. 5:**
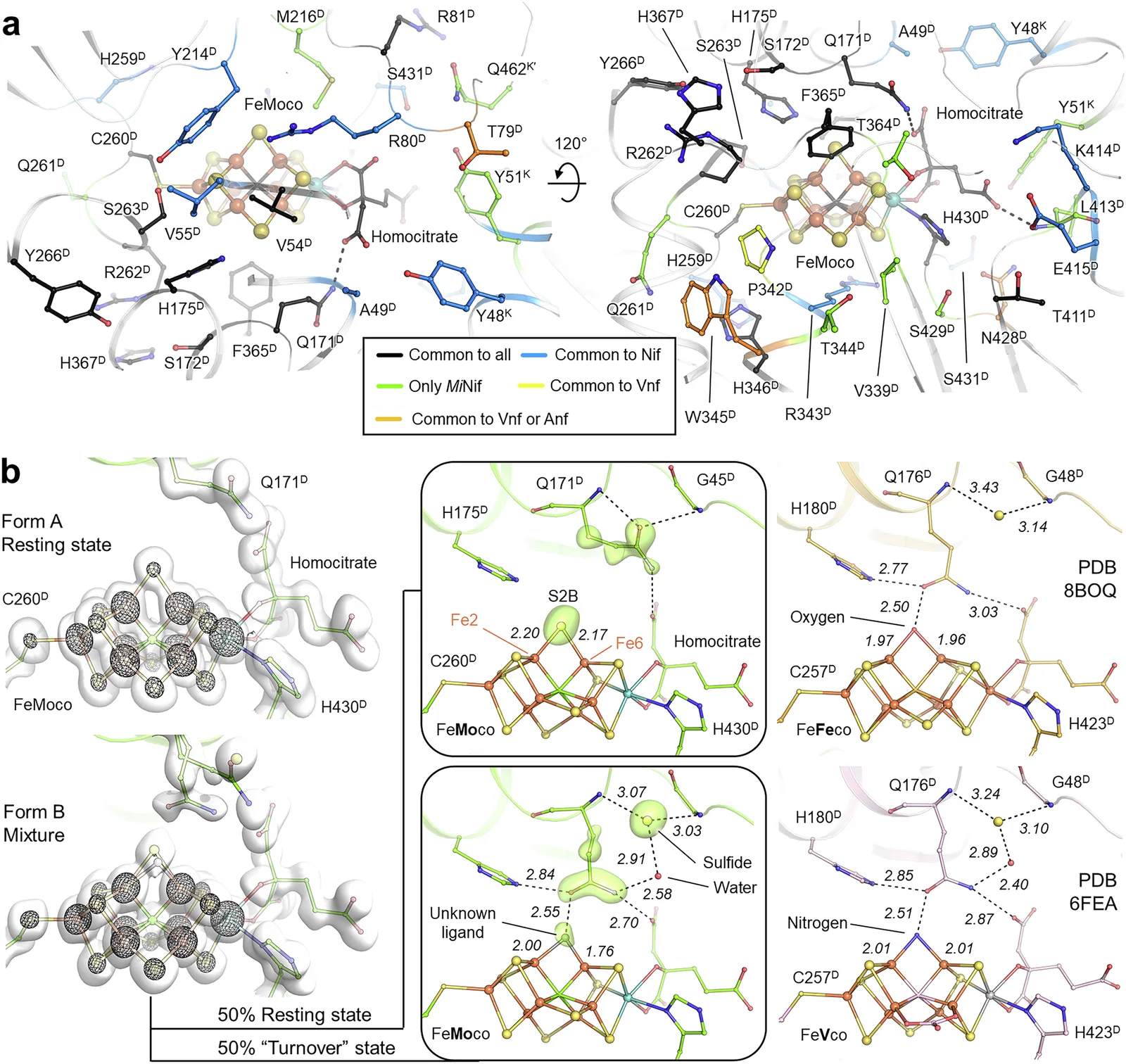
N_2_-reduction site in *Mi*NifDK. **a** Conservation of the FeMoco surroundings in *Mi*NifDK and structural homologues. The protein backbone is shown as cartoons, with residues which are part of the primary and secondary coordination spheres displayed as sticks. Metallocofactors are displayed as spheres and sticks. A colour coding of carbon shows the conservation among *Av*NifDK, *Av*VnfDK and *Av*AnfDK. Superscript´ is used to differentiate between the residue belonging to two different NifK subunits. Hydrogen bonds between the homocitrate and the protein are shown by dashed lines. **b** Close-up of the *Mi*NifDK FeMoco in the form A (top) and form B (bottom). Fe and S from the metallocofactor are displayed as spheres, and residues in the close vicinity of the FeMoco are shown as sticks. 2*F*_o_ − *F*_c_ maps are overlaid as a transparent surface (1-σ) and black mesh (9-σ). Right panels illustrate the interpretation of the mixture of different states from the form B deconvoluted as resting (top) and “turnover” (bottom) states. An omit map was generated for Gln171^D^, S2B, and the unknown ligand for the corresponding states, and the resulting difference maps are presented as a green transparent surface contoured to 6-σ. As a comparison, the FeVco/FeFeco site in the “turnover” states for *Av*VnfDK^72^, and *Av*AnfDK^52^ is presented on the right. Bond distances are indicated in Å.

The catalytic chamber residues are largely identical across all isoforms, with *Mi*NifDK specific variations occurring at the bulky Met216^D^ pointing to the molybdenum and Gln462^K^′, Tyr51^K^, Thr364^D^ slightly readjusting the water network close to the homocitrate (Fig. 5a and Supplementary Fig. 10a). The hydrogen bonding network made of Ser172^D^, His175^D^, Arg262^D^, Ser263^D^, Tyr266^D^, and His367^D^ is perfectly conserved and might be involved in proton delivery, a controversial topic^69,70^. The identity of the central ligand of the FeMoco is proposed to be a carbide atom based on the 1.21-Å structure, corroborating that the chemical structure of FeMoco is identical to that of *Av*NifDK^14,71^ (Supplementary Fig. 10b). A superposition of the metallocofactors harboured in all *A. vinelandii* isoforms confirmed the almost identical position of Fe and S atoms, as well as the homocitrate between *Mi*NifDK FeMoco and *Av*NifDK FeMoco with slight deviations within *Av*VnfDK and *Av*AnfDK (Supplementary Fig. 10c).

The form A harbours the conventional FeMoco in a “resting state” in which the S2B fully occupies its position on the FeMoco sulfur belt and the Gln171^D^ side chain binds the homocitrate and amide backbone Gly45^D^ (Fig. 5). Remarkably, the form B exhibits a mixture modelled with a 50/50 occupancy of the resting state and the so-called “turnover” state that has never been observed in a Mo-dependent nitrogenase (Fig. 5b). The turnover state was first observed in *Av*VnfDK, in which the protein preparation was obtained by diminishing the concentration of reducing agents during the cultivation^58,72^. The same mixture of states was later observed in *Av*AnfDK^52^. This state is defined by (*i*) the migration of the S2B from the sulfur belt to the position occupied by the oxo group of the Gln171^D^, (*ii*) a ligand bridging the Fe2 and Fe6, *(iii)* a repositioning of the canonical Gln171^D^ side chain binding the ligand and homocitrate, and *(iv)* a water molecule bridging the Gln171^D^ side chain and the S2B. It is worth noting that the observed movements differ from the CO or selenide-trapped structures, where the conserved glutamine was unable to rotate, and sulfide was not present^73,74^. Whether the conformation truly represents a turnover state remains a matter of debate, and it might not reflect an intermediate stage of the Lowe-Thorneley model. However, because of its usage in the literature, we kept this nomenclature in the present study. While the readjustments are the same in *Mi*NifDK, the monoatomic ligand is not equidistant between Fe2 and Fe6 as modelled in *Av*VnfDK (PDB 6FEA) and *Av*AnfDK (PDB 8BOQ) and has a shorter distance from the Fe6 (Fig. 5b). The short distance (1.76 Å) is reminiscent of Fe chemical complexes engaging a double bond with a nitrogen while remaining too large for a triple bond^75,76^. Despite the near atomic resolution, the mixture of states prevented us from providing the atom identity of the ligand as we did for the central carbide, and future studies, including spectroscopy, will have to be conducted to clarify the FeMoco status.

## Discussion

The development of methods for sustainable NH_3_ production to meet the constantly increasing demand through optimisation of N_2_ reduction technologies has become the new frontier of our modern era^6,36,77–79^. Exploiting the microbial genetic diversity of diazotrophs is a promising strategy for finding robust and more efficient nitrogenases with peculiar properties. For instance, *Mi*NifH and the enzyme involved in the FeMoco biosynthesis, *Mi*NifB, have been in the spotlight for successful transplantation *in planta*^34,35,80^. The *M. infernus* system is of particular interest for this type of biotechnological application because of its robustness and the minimal *nif* operon, reduced to four structural genes, two regulators, and three possible accessory proteins^38^. The native purification performed in this work detected only the presence of the regulator NifI_1,2_ associated with the nitrogenase^49^, suggesting that accessory proteins are not bound to *Mi*NifDK and might have a role in optimising the metallocofactor biosynthesis and enzyme maturation^38^. The growth dependency on molybdenum, with a required minimum of 100 nM MoO_4_ (Fig. 2b), alongside a remarkable tolerance to tungstate, showcases an adapted metal trafficking and recognition system that avoids tungsten misincorporation in the nitrogenase cofactor^37^. Notably, the work of Payne and colleagues^26^ showed that the minimal concentration of molybdenum necessary for diazotrophic growth in *M. infernus* may be drastically lower in its physiological environment, in which molybdenum is supposed to be rare.

Diazotrophic *Methanococcales* living in extreme environments might harbour a relic of the nitrogenase predecessor^28–30^. This theory, based on phylogenetic and the evolutionary history of methanogens, has been recently reinforced by the discovery that the reactivation system of the methanogenic enzyme (i.e., Methyl-Coenzyme M reductase) contains a metallocluster similar to an intermediate in the FeMoco biosynthesis^81^. Altogether, the fact that the biosynthesis of the catalyst for methane generation (i.e., CfbCD) requires an NifDK-like machinery reinforces the scenario that methanogens adapted these enzymes to create the primordial nitrogenase^29,30^. Our structural studies corroborate this hypothesis by highlighting structural simplifications and resemblances between *Mi*NifDK and homologues belonging to NifDK, VnfDK, and AnfDK (Fig. 3 and Supplementary Figs. 2–4), thereby suggesting that the ancestral versions of these homologues were more similar to nitrogenases from *Methanococcales*. Furthermore, the structural insights reveal the perfect conservation of metallocofactor coordination, plausible proton shuttling to the FeMoco centre and the close environment of the S2B, while some parts involved in the second metal coordination sphere, the hydrated cavity surrounding the homocitrate, and the electron transfer path could be more divergent.

*Mi*NifDK natural hyperthermostability and rigidity (Fig. 2e and Supplementary Figs. 4–6), together with the naturally reduced state of the cytoplasm in the methanogen, as well as the rapid purification and crystallisation under strict exclusion of O_2_ and dithionite, could explain why the form B retained the P^1+^ and the partial FeMoco turnover states. Trapping the P^1+^ offers a picture of the transient, electron-depleted physiological state provoked by NifH binding^16,68^. Obtaining the “turnover” state in a FeMoco-containing nitrogenase confirms the conservation of the described behaviour across all N_2_-fixing enzymes. The turnover state has been postulated to illustrate a FeMoco loaded with nitrogen-species intermediates located between the Fe2 and Fe6 following the displacement of S2B^61^, further stabilised by the canonical glutamine sidechain: a snapshot of the nitrogenase in action. However, this reaction mechanism is still challenged^82,83^, and the intrinsic characteristics of *Mi*NifDK described in this work are expected to deepen our understanding of the nitrogenase mechanism in future studies. For instance, its hyperthermostability features that hold high potential for biotechnology could also be employed to isolate new catalytic intermediates through cold trapping and characterisation by atomic-resolution crystallography and spectroscopy, providing invaluable insights for mimicry-based chemistry and translating this knowledge into the bio-inspired design of new alternatives to the current Haber-Bosch catalysts.

## Methods

### *M. infernus* growth conditions

*Mi*NifDK and *Mi*NifH were purified natively from *Methanocaldococcus infernus* DSM 11812 (Leibniz Institute DSMZ - German Collection of Microorganisms and Cell Cultures, Braunschweig, Germany). The growth medium was prepared according to Maslać et al. 2022^37^ with the following modifications: Substitution of Na_2_SeO_4_ with Na_2_SeO_3_·5H_2_O (2 µM final), the Na_2_WO_4_·H_2_O concentration was increased to 100 µM final, addition of 22 mM Na_2_SO_4_, final concentration of 10 µM Na_2_MoO_4_ and pH adjustment to 6.5. Adaptation to diazotrophic conditions was done through gradual NH_4_Cl depletion from the media by three successive transfers to the same media without NH_4_Cl, with a headspace containing N_2_ (see below), as described in Maslać et al. 2024^50^.

*M. infernus* was routinely cultivated anaerobically in 30 − 60 mL medium in 1 L pressure-resistant Duran bottles with 0.25 to 2 mM final concentration of Na_2_S as a reductant and sulfur source, with a preference of 0.5 mM Na_2_S final in molybdate-containing cultures. After vacuuming the headspace of the bottle to − 1 bar, it was filled to reach 0.5 bar H_2_/CO_2_ (80:20%) and complemented to 1 bar final with 100% N_2_. The inoculum was added in a 1:20 ratio. The cultures were incubated standing without agitation at 75 °C for two days. After roughly 24 h, the gas phase was exchanged by vacuuming ( − 1 bar) followed by filling up to 0.5 bar H_2_/CO_2_ (80:20%) and completing to 1 bar final with 100% N_2_ gas. Cells were harvested at room temperature in an anaerobic chamber containing a gas mixture of N_2_/CO_2_ (90:10) via centrifugation at 6000 x *g* at 20 °C for 30 min. The wet cell pellet was transferred to a sealed serum flask, 0.8 bar of N_2_ was added, and the flask was stored at − 80 °C. All proteins studied in this work come from batch cultures.

*M. infernus* could also be grown in a fermenter, as presented in Fig. 2c. Here, biomass production of *M. infernus* was carried out in a 10 L fermenter (BIOSTAT® B plus, Sartorius, Göttingen, Germany) using 7 L of the same culture medium. Anoxic conditions were achieved by sparging the fermenter with N_2_ before inoculation for a few hours. Pre-cultures grown as described above were used as inoculum at a 1:20 ratio. The fermenter was maintained at 75 °C, and 300 μL of Antifoam 204 (Sigma Aldrich, St. Louis, USA) was added immediately before inoculation to prevent foaming. The redox balance and sulfur sources were maintained by supplying a 1.6 M stock of Na_2_S solution continuously, with an initial flow rate of 1.5 mL.min^−1^, which was increased to 3 mL.min^−1^ after 11 h. The culture was continuously sparged with H_2_/CO_2_ (80:20%) at 2 L.min^−1^ and N_2_ at 0.5 L.min^−1^, with stirring at 500 rpm. After 11 h, the gas supply was increased to 5 L.min^−1^ for H_2_/CO_2_ and 1 L.min^−1^ for N_2_, while the stirring speed was raised to 600 rpm. The cultivation continued for 6.5 h and was stopped at an OD_600nm_ of ≈ 0.89. *M. infernus* cells grown in the fermenter were harvested under anaerobic conditions by using an adapted 2 L glassware to collect the cells and transfer them to the anaerobic chamber containing a gas atmosphere of N_2_/CO_2_ (90:10%). The cells were centrifuged at 6000 x *g* at 20 °C for 20 min. Pellets were transferred to serum flasks, supplemented with 0.8 bar of H_2_/CO_2_ (80:20%) and stored at − 80 °C.

For the physiological experiments testing different temperatures, vanadium addition, molybdate concentrations, and N_2_ pressure, a culture volume of 10 mL was used in 1 L pressure-resistant Duran bottles. Regarding molybdate depletion, several transfers without molybdenum with a limited amount of NH_4_Cl (22 mM final) were made, and the cultures were switched to diazotrophy with insufficient leftover ammonium to promote growth. In this case, molybdate was excluded from the trace elements and added anaerobically directly to the medium. The volume of the inoculum was 1:10 except for the N_2_-dependent growth on Supplementary Fig. 1b, where it was 1:20. The same temperature or medium composition was utilised except for the mentioned varying conditions. Experiments were performed in triplicate with a final sodium sulfide concentration of 0.25 − 0.5 mM. For the N_2_-dependent growth experiment, different volumes of 100% N_2_ were injected into a 1 L bottle containing 1 bar of H_2_/CO_2_: 0.5 mL (0.02 mmol, 0.00049 atm), 5 mL (0.2 mmol, 0.0049 atm), or 50 mL (2.0 mmol, 0.049 atm). The bottle contained 20.2 mmol N_2_ (0.49 atm) when 0.5 bar of N_2_ was present in the bottle as described above. Partial pressures were calculated based on the ideal gas law, assuming ideal behaviour for N_2_. All values were calculated under standard conditions (i.e., 25 °C). Notably, the moles of N_2_ and N_2_ partial pressure in atm displayed represent the total quantity in the bottles, not how much N_2_ would be dissolved at 75 °C. The cultures were incubated for 48 h and were sampled five times.

### Protein purification

The *Mi*NifDK and *Mi*NifH were purified several times with similar protocols. A representative purification protocol is described below. The reproducible purification procedure of both proteins was controlled by SDS-PAGE, absorbance at 280, 415, and 550 nm, and visual inspection of the brown colour of the proteins.

An 11.8 g wet weight of cells was processed. Frozen cell pellets were transferred to an anaerobic chamber containing a gas atmosphere of N_2_/CO_2_ (90:10%) at room temperature. 60 mL of lysis buffer (50 mM Tris/HCl, pH 8.0, 2 mM dithiothreitol (DTT)) was added to lyse the cells through osmotic shock. The viscous lysate was sonicated using a KE76 probe Bandelin SONOPULS (Sigma, Berlin, Germany) at 75% power four times, 30 s each. The resulting lysate was centrifuged at 45,000 x *g* for 45 min at 20 °C. After centrifugation, the supernatant was transferred to a Coy tent containing a gas atmosphere of N_2_/H_2_ (97:3%) under yellow light and filtered through a 0.2 μm filter (Sartorius, Göttingen, Germany). Filtered supernatant was loaded onto a 5 mL HiTrap^TM^ Q Sepharose High-Performance column (GE Healthcare Life Sciences, Munich, Germany), pre-equilibrated in lysis buffer at 3 mL.min^−1^. Proteins were eluted with a 0.2 to 0.5 M NaCl linear gradient in lysis buffer in 45 minutes with a 1 mL.min^−1^ rate (9 column volumes, CV). *Mi*NifDKI_1,2_ eluted mainly between 0.41 and 0.47 M NaCl. Pooled fractions were diluted 1:1 with lysis buffer supplemented with 2 M (NH_4_)_2_SO_4_ and filtered again with a 0.2 μm filter (Sartorius, Göttingen, Germany). The filtrate was loaded onto a Source^TM^ 15PHE 4.6/100 PE column (Cytiva, Freiburg, Germany) pre-equilibrated with lysis buffer supplemented with 2 M (NH_4_)_2_SO_4_ to perform hydrophobic interaction chromatography. After washing the column, proteins were eluted with a 1 M to 0.34 M (NH_4_)_2_SO_4_ linear gradient for 15 minutes at a 1 mL.min^−1^ flow rate (8.8 CV). Fractions containing *Mi*NifDKI_1,2_ eluted between 0.74 and 0.34 M (NH_4_)_2_SO_4_. Pooled fractions were concentrated to 5 mL using a 50-kDa cut-off Vivaspin centrifugal concentrator (Sartorius, Göttingen, Germany). The concentrate was diluted with 25 mL lysis buffer containing freshly prepared 10 mM 2-oxoglutarate, 2 mM MgCl_2_, and 2 mM ATP and incubated overnight inside the Coy tent to separate *Mi*NifDK from *Mi*NifI_1,2_. The sample was filtered through a 0.2 μm filter and loaded onto a 5 mL HiTrap^TM^ Q Sepharose High-Performance column (GE Healthcare Life Sciences, Munich, Germany) pre-equilibrated with the same lysis buffer containing the ligands at the same concentration. After washing, the protein was eluted with a 0.15 to 0.4 M NaCl linear gradient in the same ligand-supplemented buffer in 20 minutes at a 1 mL.min^−1^ flow rate (4 CV). Fractions containing only *Mi*NifDK eluted between 0.34 and 0.39 M NaCl. The sample was then concentrated with a 50-kDa cut-off Vivaspin centrifugal concentrator, and the buffer was exchanged by ultrafiltration for the storage buffer (25 mM Tris/HCl pH 8.0, 10% v/v glycerol, 100 mM NaCl, and 2 mM DTT).

*Mi*NifH purification was carried out with the same cell preparation. *Mi*NifH mainly eluted between 0.36 and 0.4 M NaCl during the first anionic exchange chromatography step. This pool was diluted with three volumes of the lysis buffer supplemented with 2 M (NH_4_)_2_SO_4_, filtered through a 0.2 μm filter and loaded on a 5 mL Phenyl Sepharose^TM^ High-Performance column (GE Healthcare Life Sciences, Munich, Germany) pre-equilibrated with the lysis buffer supplemented with 2 M (NH_4_)_2_SO_4_. After column washing, proteins were eluted with a 1.5 to 0.5 M (NH_4_)_2_SO_4_ linear gradient for 75 minutes with a 1 mL.min^−1^ flow rate (15 CV). *Mi*NifH eluted between 1.1 and 0.84 M (NH_4_)_2_SO_4_. Fractions were pooled, and the buffer was exchanged for lysis buffer by ultrafiltration on a 30-kDa cut-off Vivaspin centrifugal concentrator (Sartorius, Göttingen, Germany). The resulting sample was filtered and injected on a 5 mL HiTrap^TM^ Q Sepharose High-Performance column pre-equilibrated with lysis buffer. After column washing, the elution was performed by applying a 0 to 75% linear gradient of 500 mM sodium malate pH 5.4 and 2 mM DTT for 25 min with a 1.5 mL.min^−1^ flow rate (7.5 CV, lysis buffer being the second buffer for the elution). *Mi*NifH eluted between 50 and 62% of 500 mM sodium malate, pH 5.4 and 2 mM DTT. The pooled sample was concentrated with a 30-kDa cut-off Vivaspin centrifugal concentrator (Sartorius, Göttingen, Germany), and the buffer was exchanged for the storage buffer.

### Protein denaturation experiment

To measure the melting point of as-isolated *Mi*NifDK, a denaturation experiment was performed under the same conditions as those in Supplementary Fig. 3 of Maslać et al., 2024, where the assay is downscaled to a 20 µL volume reaction^50^. The experiment was performed in duplicate in a Coy anaerobic chamber (Coy Laboratory Products) containing a N_2_/H_2_ gas atmosphere (97:3%). *Mi*NifDK was diluted to a final concentration of 5 mg.mL^−1^ in 50 mM Tris/HCl pH 8.0, 100 mM NaCl, and 2 mM DTT in a volume of 20 μL. Eppendorf tubes containing *Mi*NifDK were incubated for 25 min at different temperatures in a block heater. After incubation, the tubes were immediately centrifuged for 25 min at 6000 x *g* at 20 °C. 5 μL of each supernatant was diluted 10 times with 50 mM Tris/HCl, pH 8.0 and 2 mM DTT before estimation of the protein concentration by the Bradford method, performed in aerobic conditions and using bovine serum albumin (BSA) as standard. The values reported in the graphs of Fig. 2e correspond to the protein quantification ratios of the supernatants before and after heat treatment. The obtained values were compared with those reported in the work of Maslać et al., 2024^50^ for *Av*NifDK.

### UV/Visible spectroscopy

As-isolated *Mi*NifDK was transferred inside an anaerobic chamber filled with a 100% N_2_ atmosphere. Absorbance spectra were recorded at room temperature in a FLUOstar Omega Microplate reader (BMG Labtech, Germany) in 384-well plates. The spectra were measured using 15 µL of 0.3 mg.mL^−1^ *Mi*NifDK prepared in storage buffer, supplemented or not with 5 µL of a 0.5 mM dithionite solution (0.225 mg.mL^−1^ *Mi*NifDK and 0.125 mM dithionite final). Blanks were measured in storage buffer or in the storage buffer supplemented with 0.125 mM dithionite. Absorbance values for both samples were blank-subtracted, and values obtained for *Mi*NifDK with 0.125 mM dithionite were rationalised by multiplying values by a 1.333 factor.

### Activity measurements

Activity assays were carried out as described in Maslać et al. 2024^50^ with a few adjustments in volume, time, and temperature. The final reaction volume was 100 µL, incubated for 30 min at 50 °C. Experiments were done in an anaerobic glovebox filled with a gas phase of 100% N_2_. A freshly prepared sodium dithionite solution was used as an electron donor to the system. A *Mi*NifDK:*Mi*NifH molar ratio of 1:16 was used. The reaction mixture contained 100 mM MOPS buffer at pH 7.0, 30 mM phosphocreatine, 5 mM ATP, 0.6 mg.mL^−1^, BSA, 0.2 mg.mL^−1^, creatine phosphokinase (from Rabbit muscle, Merck/Roche, Darmstadt, Germany), 10 mM MgCl_2_, 10 mM dithionite, 0.1 mg.mL^−1^ *Mi*NifDK and 0.5 mg.mL^−1^ *Mi*NifH. All reactants and proteins were prepared in 100 mM MOPS buffer, pH 7.0. Traces of (NH_4_)_2_SO_4_ coming from the *Mi*NifH purification generate a background that was subtracted to calculate *Mi*NifDK-specific activity (Fig. 2f).

For the *Mi*NifDKI_1,2_ response to 2-oxoglutarate, the pool that eluted from the Source^TM^ 15PHE 4.6/100 PE column was further purified. Two protocols can be used to enrich the protein. In the first one, the pool was diluted with three volumes of lysis buffer, filtered through a 0.2 μm filter and loaded on a 5 mL HiTrap Q Sepharose^TM^ High-Performance column (GE Healthcare Life Sciences, Munich, Germany) pre-equilibrated with the same buffer. After washing, the elution was performed by applying a 0 to 75% linear gradient of 500 mM sodium malate, pH 5.4 and 2 mM DTT, resulting in the enrichment of *Mi*NifDKI_1,2_ in fractions collected between 50 and 62% of the gradient (lysis buffer being the second buffer used for the gradient). In the second protocol, the pool from the Source^TM^ 15PHE 4.6/100 PE column was further purified and supplemented with a final concentration of 1 M (NH_4_)_2_SO_4_, filtered through a 0.2 μm filter and loaded on a 5 mL Phenyl Sepharose^TM^ High-Performance column (GE Healthcare Life Sciences, Munich, Germany) pre-equilibrated with the lysis buffer supplemented with 1 M (NH_4_)_2_SO_4_. A gradient from 1 to 0 M (NH_4_)_2_SO_4_ was performed, resulting in the enrichment of *Mi*NifDKI_1,2_ in fractions collected between 0.54 and 0.34 M of the gradient. The pooled sample was then concentrated through a 30-kDa cut-off Vivaspin centrifugal concentrator (Sartorius, Göttingen, Germany) and injected on a Superose 6 Increase 10/300 (GE Healthcare Life Sciences, Munich, Germany) after removal of particles through centrifugation or filtering. Elution was performed at a flow rate of 0.4 mL.min^−1^ in the storage buffer containing 150 mM NaCl final. The pooled sample was finally concentrated through a 30-kDa cut-off Vivaspin centrifugal concentrator.

The assay was performed with a final amount of 0.008 mg *Mi*NifDK per assay, accounting for a molar ratio of 1 mol *Mi*NifDKI_1,2_:0.8 mol of *Mi*NifDK. The activity presented in the Supplementary Fig. 1c is relative to the highest activity measured when the complex recovers full activation at 10 mM 2-oxoglutarate.

Since no reactions were measurable at room temperature in the glovebox (18 − 20 °C, Fig. 2f), we defined the start of the reaction when the reaction mixtures were placed on a block heater. After an incubation at 50 °C for 30 min, the reaction was quenched using 30 µL of 400 mM EDTA (adjusted to pH 8.0). NH_3_ measurement was carried out aerobically with the o-phthalaldehyde method (described below^84–86^), directly or after overnight storage at 4 °C. We previously determined that under these conditions, *Mi*NifDKH activity is linear in the first 40 min. Omission of one component/substrate/cofactor was used as a negative control, and three replicates were used for each condition.

NH_3_ measurement was carried out using a solution of 100 mL 0.2 M phosphate buffer pH 7.0 containing 2.7 mg.mL^−1^ o-phthalaldehyde, 3.5 mM 2-mercaptoethanol and 2% (v/v) ethanol. This reagent mixture was prepared in the dark, where o-phthalaldehyde was first dissolved in 5 mL of pure ethanol, and then added to the phosphate buffer solution alongside the 2-mercaptoethanol. 25 µL of standard or sample was added to 1 mL of the o-phthalaldehyde reagent mixture. A brief vortexing step was used to homogenise the samples in Eppendorf tubes. 300 µL of the mixture was transferred into a black 96-well plate (FALCON, Germany) in 3 replicates (3 × 300 µL). The plate was covered with aluminium foil and incubated in the dark for 30 min. Afterwards, fluorescence was measured using excitation at 410 nm and by measuring emission at 472 nm. Different concentrations of NH_4_Cl solutions were used as standards.

### Crystallisation and structure determination

After purification, samples were centrifuged at 13,000 x *g* for 3 min to remove macro-aggregates and dust, and crystallised inside an anoxic chamber filled with a N_2_/H_2_ (97:3%) atmosphere and maintained at 20 °C. Crystallisation was done by the sitting drop method in 96-well MRC 2-drop polystyrene crystallisation plates (SWISSCI). The crystallisation drop was formed by spotting 0.7 μL of purified protein with 0.7 μL of precipitant. Both crystalline forms were obtained using the same crystallisation condition made of 30% v/v 2-methyl-2,4-pentanediol, 100 mM Tris pH 8.5, 500 mM NaCl and 8% w/v Polyethylene glycol 8000 (crystallisation solution of the JBScreen Wizard from Jena Bioscience, Germany).

*Mi*NifDK crystals form A were obtained with a protein solution at a final concentration of 2.95 mg.mL^−1^. *Mi*NifDK crystals form B were obtained by the same method and crystallisation solution, except that the protein was crystallised at 22.4 mg.mL^− 1^.

Crystals of form A took about a month to appear, while the ones from form B grew after a few days and were harvested in a short time following their appearance. Both crystals were directly flash-frozen in liquid nitrogen.

### Data collection and structural analysis

The diffraction experiments used for the deposited model were performed at 100 K on beamlines X06-SA PXI at the Swiss Light Source synchrotron (SLS) for form A and BM07-FIP2 at the European Synchrotron Radiation Facility (ESRF) for form B. The data were processed and scaled with *autoPROC*^87^. The data presented slight anisotropy, and hence we used the *STARANISO* correction integrated with the *autoPROC* pipeline^88^ (*STARANISO*. Cambridge, United Kingdom: Global Phasing Ltd.). Datasets of form B collected at 19.9 and 20.1 keV were processed without *STARANISO* correction.

The structure of form A was solved by using a computationally generated AlphaFold 2 model as a template of the *Mi*NifDK complex for molecular replacement^89^. The structure of form B was solved by using the model of form A as a template for molecular replacement. In both cases, molecular replacement was done with *PHASER* from the *Phenix* package^90^. Models were manually built via *Coot*^91^ and refined with *phenix.refine*^92^. The refinement steps were performed by considering all atoms as anisotropic with hydrogens in the riding position. Models were validated by the MolProbity server^93,94^. Data collection and refinement statistics for the deposited models are listed in Supplementary Table 1. It must be noted that models were deposited with hydrogens, as they significantly affect the final R-factor and R-free. Form A at 1.37 Å has a Rfactor/Rfree of 12.33/15.56% with modelled hydrogens and 13.40/16.35 % without hydrogens. Form B at 1.21 Å has a Rfactor/Rfree of 12.08/14.60% with modelled hydrogens and 13.27/16.56% without hydrogens.

All figures with structures were generated and rendered with PyMOL (Version 2.2.0, Schrödinger, LLC, New York, NY, United States).

A total of four datasets were used to generate the maps in Fig. 3a. The grey-blue density map highlighting the molybdenum atom was created by first generating anomalous maps at 19.9 keV (Mo K-edge remote) and 20.1 keV (Mo K-edge peak) (Supplementary Table 1). An anomalous difference Fourier map was generated by subtracting the anomalous map at 20.1 keV from the one at 19.9 keV. The signal was finally exemplified by generating a non-crystallographic symmetry (NCS) map from this difference map. The same procedure was applied for the Fe, in which the difference map resulted from subtracting the anomalous map generated at 7.16 keV (Fe K-edge peak) from the one generated at 6.00 keV (Fe K-edge remote). Note that no signals can be visible for the third metal site, in which magnesium was modelled based on its adequate B-factor.

Sequence and structural comparisons to generate graphs and pictures of Figs. 3 to 5, and for the Supplementary Figs. 2–7, 10 were done with the following models: *A. vinelandii* NifDK (*Av*Nif) PDB code 3U7Q^14^, *A. vinelandii* VnfDK (*Av*Vnf) PDB code 5N6Y^58^, *A. vinelandii* AnfDK (*Av*Anf) PDB code 8BOQ^52^, *Klebsiella pneumoniae* NifDK (*Kp*Nif) PDB code 1QH8^65^, *Clostridium pasteurianum* NifDK (*Cp*Nif) PDB code 4WES^95^, *Gluconacetobacter diazotrophicus* PA1 5 NifDK (*Gd*Nif) PDB code 5KOH^66^, and *Rhodobacter capsulatus* AnfDK (*Rc*Anf) PDB code 8OIE^62^.

In Fig. 3b, the sequence alignment superposed on the secondary structure of *Mi*NifDK is presented in the Supplementary Figs. 2, 3. The latter were made with the Espript server^96^.

### Reporting summary

Further information on research design is available in the Nature Portfolio Reporting Summary linked to this article.

## Supplementary information

Supplementary information

Reporting Summary

Transparent Peer Review file

## Source data

Source Data

## Data Availability

The raw data generated in this study to generate Fig. 2 and Supplementary Fig. 1 can be found in the Source Data file. *Mi*NifDK structures were validated and deposited in the Protein Data Bank (PDB) under the following accession numbers: 9SPZ https://doi.org/10.2210/pdb9SPZ/pdb, Crystal structure of the nitrogenase from *Methanocaldococcus infernus* refined to 1.37 Å resolution, form A. 9SQ3 https://doi.org/10.2210/pdb9SQ3/pdb, Crystal structure of the nitrogenase from *Methanocaldococcus infernus* refined to 1.21 Å resolution, form B. Source data are provided in this paper.
