# Supplementary material for: Molecular basis of N2 fixation in a hyperthermophilic archaeon
Source: Nat Commun. 2026 Sep 8;17:9602. doi: 10.1038/s41467-026-77173-0 (PMC13554218; doi:10.1038/s41467-026-77173-0)
Supplement: Supplementary file 1 [file 41467_2026_77173_MOESM1_ESM.pdf]

Molecular basis of N_2 fixation in a hyperthermophilic archaeon

Nevena Maslać, Mustafa Rasim Törer[ϕ], Pauline Bolte[ϕ], and Tristan Wagner[*]

[ϕ] Equally contributed.

Supplementary information.

This file contains one table and ten figures:

Supplementary Table 1. Data collection and refinement statistics.

Supplementary Figure 1: Physiological capability of diazotrophic *M. infernus*, isolation of *Mi*NifH and partially oxidised *Mi*NifDK.

Supplementary Figure 2: Sequence and structural alignment of *Mi*NifD with homologues.

Supplementary Figure 3: Sequence and structural alignment of *Mi*NifK with homologues.

Supplementary Figure 4: Structural reduction of *Mi*NifDK compared to homologues.

Supplementary Figure 5: *Mi*NifDK composition in terms of hydrophobic clusters, glycine and proline and comparison to *Av*NifDK.

Supplementary Figure 6: Hot spots in *Mi*NifDK.

Supplementary Figure 7: Comparison of the P-cluster binding in different models and omit maps for the FeMoco.

Supplementary Figure 8: Omit map for all P-cluster states in forms A and B

Supplementary Figure 9: Superposition of form A and B, highlighting the spatial conservation of the residues

Supplementary Figure 10: Comparison of the water network surrounding the homocitrate and comparison in the carbide-containing FeMoco.

References

Supplementary Table 1. Data collection and refinement statistics.

	***Mi*NifDK form A 12.398 keV**	***Mi*NifDK form A 6.000 keV**	***Mi*NifDK form A 7.160 keV**	***Mi*NifDK form B 12.658 keV**	***Mi*NifDK form B 19.900 keV**	***Mi*NifDK form B 20.100 keV**
**Data collection**						
Wavelength (Å)	1.00000	2.06639	1.73160	0.97951	0.62304	0.61684
Space group	$P2_1$	$P2_1$	$P2_1$	$P1$	$P1$	$P1$
Resolution (Å)	78.85 – 1.37 (1.52 – 1.37)	78.99 – 2.24 (2.50 – 2.24)	78.94 – 1.84 (2.03 – 1.84)	101.86 – 1.21 (1.35 – 1.21)	102.00 – 1.81 (1.84 – 1.81)	70.73 – 2.00 (2.03– 2.00)
Cell dimensions						
a, b, c (Å)	78.43, 117.18, 106.63	78.56, 117.72, 106.56	78.55, 117.58, 106.56	68.50, 80.24, 105.74	68.60, 80.35, 105.89	68.54, 80.27, 105.80
α, β, γ (°)	90, 91.48, 90	90, 91.58, 90	90, 91.60, 90	74.49, 82.25, 65.02	74.49, 82.25, 65.02	74.50, 82.24, 65.0
R_{merge}(%)[a]	9.1 (119.8)	20.3 (58.0)	9.8 (53.3)	7.4 (107.1)	17.8 (163.3)	22.2 (167.9)
R_{pim} (%)[a]	2.9 (40.4)	14.5 (47.0)	6.9 (45.4)	3.0 (42.5)	17.8 (163.3)	22.2 (167.9)
$CC_{1/2}$[a]	0.998 (0.658)	0.984 (0.713)	0.996 (0.724)	0.999 (0.669)	0.980 (0.352)	0.979 (0.325)
I/σ_I[a]	12.3 (1.9)	5.6 (1.6)	8.9 (1.7)	12.9 (1.7)	3.5 (0.5)	2.2 (0.4)
Spherical compl. (%)[a]	68.0 (13.0)	66.8 (11.9)	74.0 (14.2)	62.1 (11.0)	93.0 (94.1)	92.5 (92.9)
Ellipsoidal compl. (%)[a]	95.3 (68.2)	91.8 (61.8)	93.3 (57.1)	92.0 (74.5)	/	/
Redundancy[a]	10.4 (9.6)	5.5 (4.6)	5.4 (4.3)	7.1 (7.3)	3.8 (3.8)	3.8 (3.8)
Nr. unique reflections[a]	273,586 (13,675)	62,114 (3,107)	124,152 (6,209)	371,908 (18,595)	168,457 (8,470)	122,976 (6,176)
**Refinement**						
Resolution (Å)	21.91 – 1.37			33.62 – 1.21		
Number of reflections	273,457			371,077		
R_{work}/R_{free}[b] (%)	12.33/15.56			12.08/14.60		
Number of atoms[c]	16,851			17,736		
Protein[c]	15,164			15,422		
Ligands/ions[c]	233			291		
Solvent[c]	1,454			2,023		
Mean B-value ($Å^2$)	23.64			17.71		
Molprobity clash score, all atoms	2.15			2.29		
Ramachandran plot						
Favoured regions (%)	96.88			97.15		
Outlier regions (%)	0			0		
rmsd[d] bond lengths (Å)	0.011			0.013		
rmsd[d] bond angles (°)	1.262			1.472		
**PDB ID code**	9SPZ			9SQ3		

[a] Values relative to the highest resolution shell are within parentheses. [b] R_{free} was calculated as the R_{work} for 5 % of the reflections that were not included in the refinement. [c] Hydrogens are excluded from the numbers of atoms. [d] rmsd, root mean square deviation. Compl. Stands for Completeness.

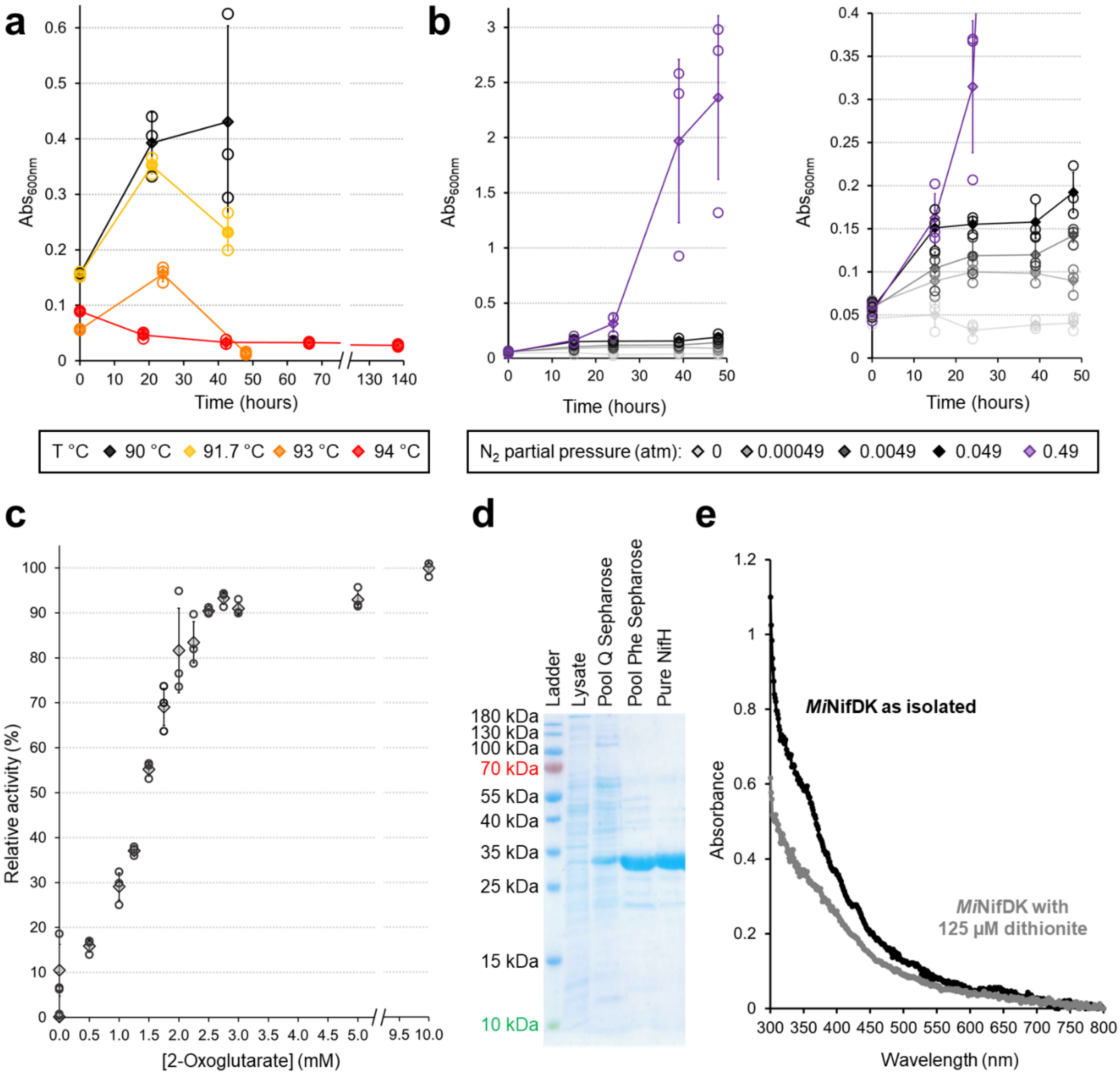

Supplementary Fig. 1. Physiological capability of diazotrophic *M. infernus*, isolation of *Mi*NifH and partially oxidised *Mi*NifDK. a, Growth curves of *M. infernus* grown diazotrophically at different temperatures (n=3). The slight increase in Abs_{600nm} at 93 °C was not considered as growth. Diazotrophic precultures for the experiment at 93 °C and 94 °C were first adapted to 90 °C before inoculation. **b,** Growth curves of *M. infernus* (n=3) with different N_2 amounts (0, 0.02, 0.2, 2, 20.2 mmol N_2 added in 1 L). Partial pressures were calculated at standard conditions (i.e., 25 °C), assuming ideal behaviour for N_2. The right panel is a close-up of the low N_2 addition. **c,** 2-oxoglutarate-dependent inhibition of *Mi*$NifI_{1,2}$ relative to the maximum activity recorded at 10 mM 2-oxoglutarate (n=3). The NH_3-production activity assay was performed by combining *Mi*$NifDKI_{1,2}$ with *Mi*NifH at 50 °C under 100% N_2 at pH 7.0 for 30 min. A $K_{0.5}$ of 1.425 mM was estimated by testing a 0 to 10 mM 2-oxoglutarate range. A control without ATP and containing 10 mM 2-oxoglutarate did not provide a signal for NH_3 production (relative activity of 0.1 ± 0.6 % compared with added ATP). **d,** Native purification of *Mi*NifH used for the activity assay. In this case, the last chromatography step contains $(NH_4)_2SO_4$, and despite intense washing through ultrafiltration, the purified NifH fraction was still contaminated with NH_4^+, explaining the background observed in Fig. 2f. **e,** UV/visible spectrophotometry profile of as-isolated *Mi*NifDK before and after addition of dithionite (n=1, 0.225 $mg.mL^{-1}$ *Mi*NifDK and 125 µM dithionite final, see Materials and Methods).

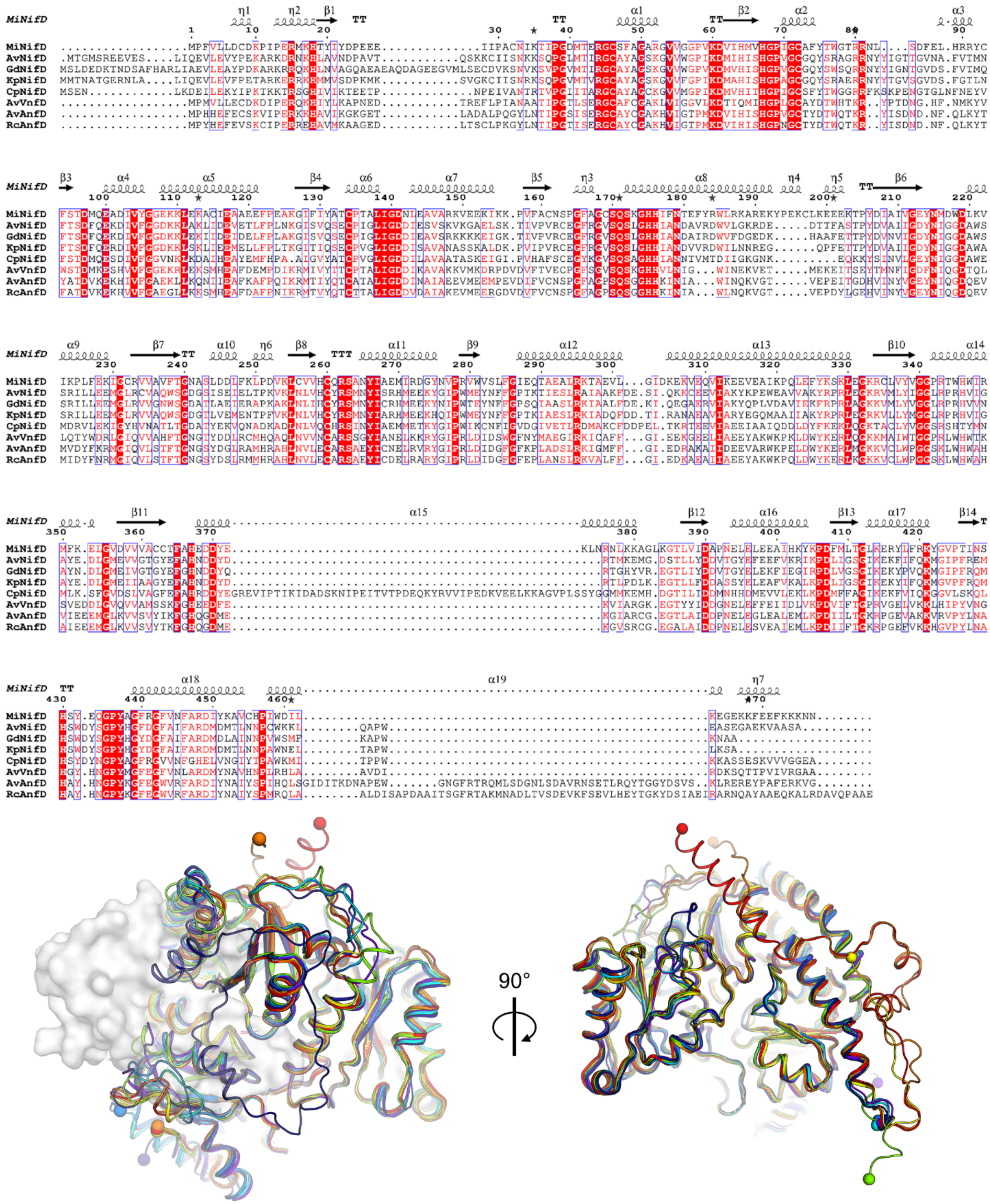

Supplementary Fig. 2. Sequence and structural alignment of *Mi*NifD with homologues. The secondary structure derived from *Mi*NifD with the sequence alignment was generated with the Espript server[1]. The bottom panel represents two views of NifD, with the protein backbone shown as cartoons. The white surface corresponds to *Av*VnfG. Structures are colored as follows: *Mi*NifD form B (green), *Av*NifD (cyan), *Gd*NifD (purple), *Kp*NifD (blue), *Cp*NifD (dark blue), *Av*VnfD (yellow), *Av*AnfD (orange), *Rc*AnfD (red).

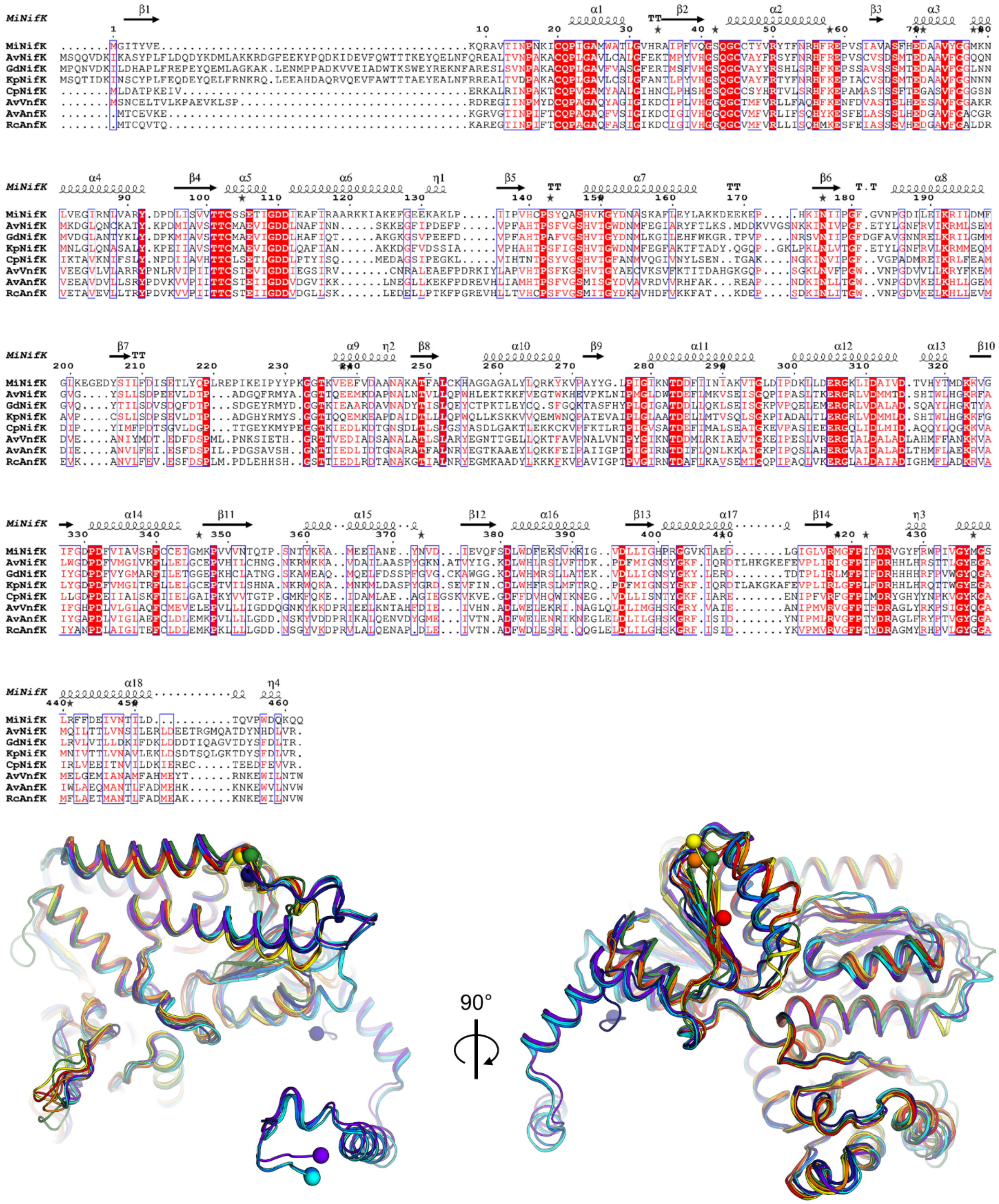

Supplementary Fig. 3. Sequence and structural alignment of *Mi*NifK with homologues. The secondary structure derived from *Mi*NifK with the sequence alignment was generated with the Espript server[1]. The bottom panel represents two views of NifK with the protein backbone shown as cartoons. *Mi*NifK form B (dark green), *Av*NifK (cyan), *Gd*NifK (purple), *Kp*NifK (blue), *Cp*NifK (dark blue), *Av*VnfK (yellow), *Av*AnfK (orange), *Rc*AnfK (red).

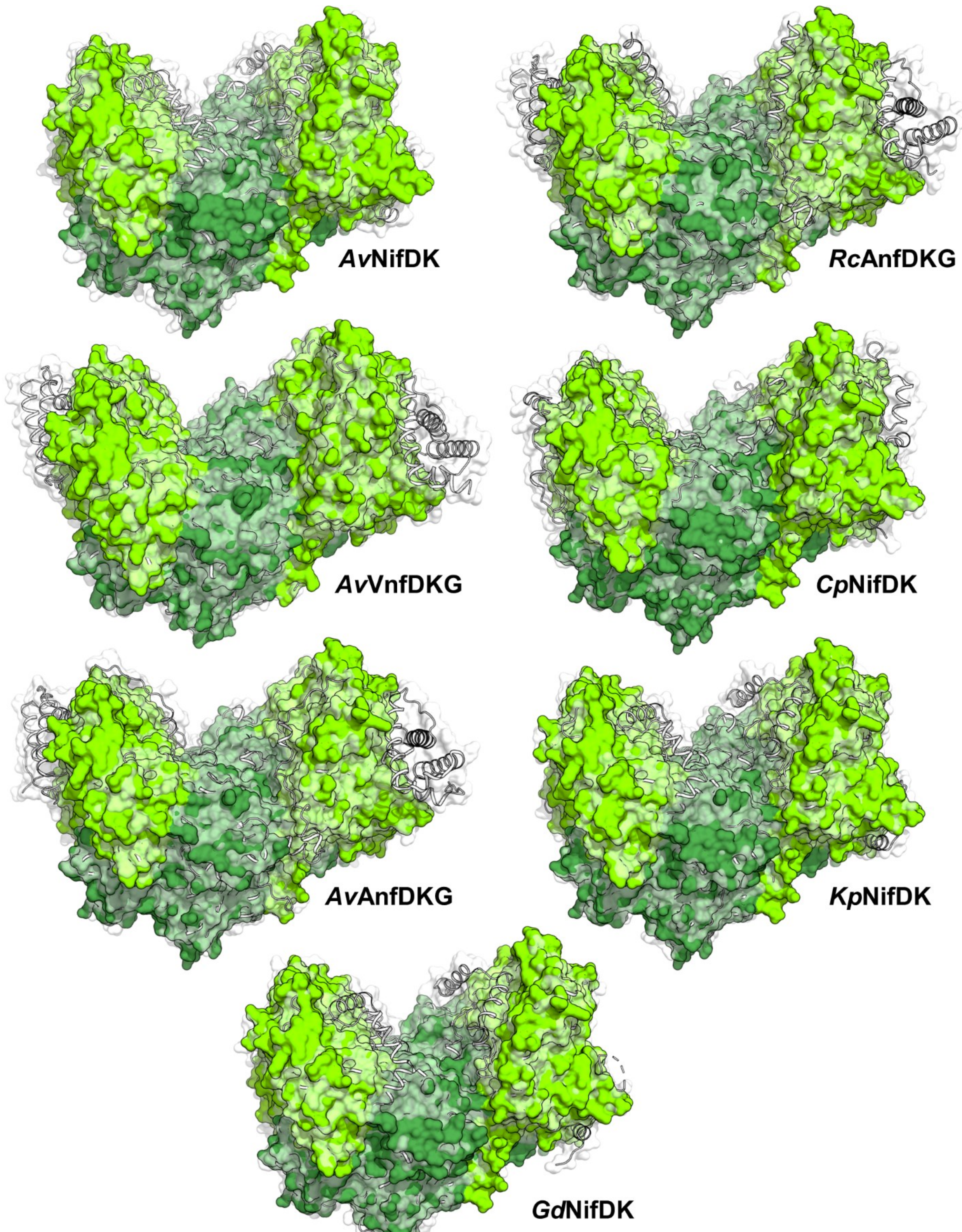

Supplementary Fig. 4. Structural reduction of *Mi*NifDK compared to homologues. *Mi*NifD (light green) and *Mi*NifK (dark green) are shown as plain surfaces with superposed structural homologues displayed as a white transparent surface and white cartoons. *Av*VnDKG comparison is slightly tilted compared to the others for better observation of the secondary elements.

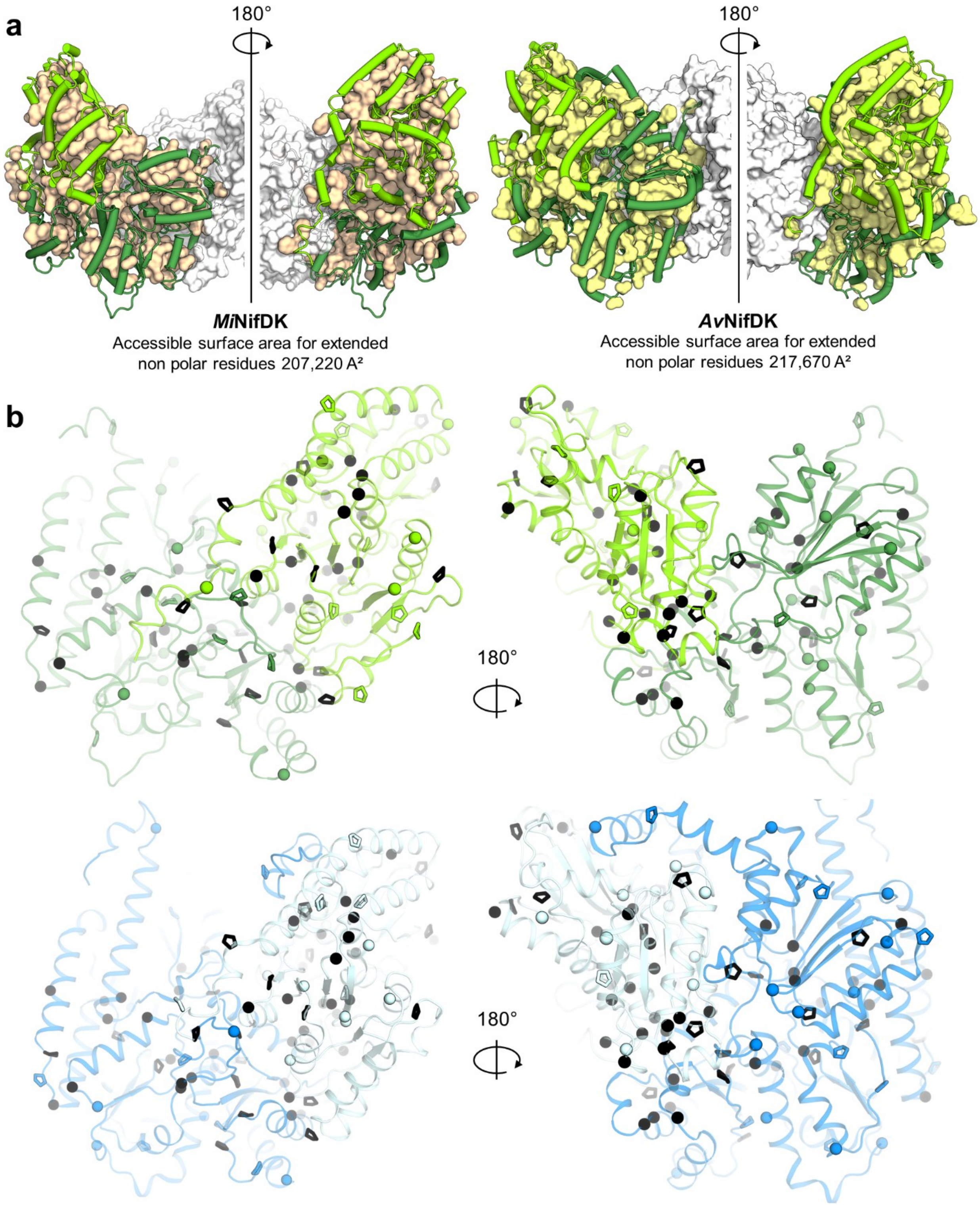

Supplementary Fig. 5. *Mi*NifDK composition in terms of hydrophobic clusters, glycine and proline and comparison to *Av*NifDK. a, Hydrophobic clusters (side chains of Val, Leu, Met, and Phe) in *Mi*NifDK (left) and *Av*NifDK (right, PDB 3U7Q). One NifDK is shown as a cartoon, and the second one as a white surface. The hydrophobic clusters are shown as a wheat/yellow surface. **b,** *Mi*NifD (light green), *Mi*NifK (dark green), *Av*NifD (cyan) and *Av*NifK (blue) are shown as cartoons with glycine Cα displayed as spheres and proline as sticks. Glycine and proline residues shared between *Mi*NifDK and *Av*NifDK are colored in black.

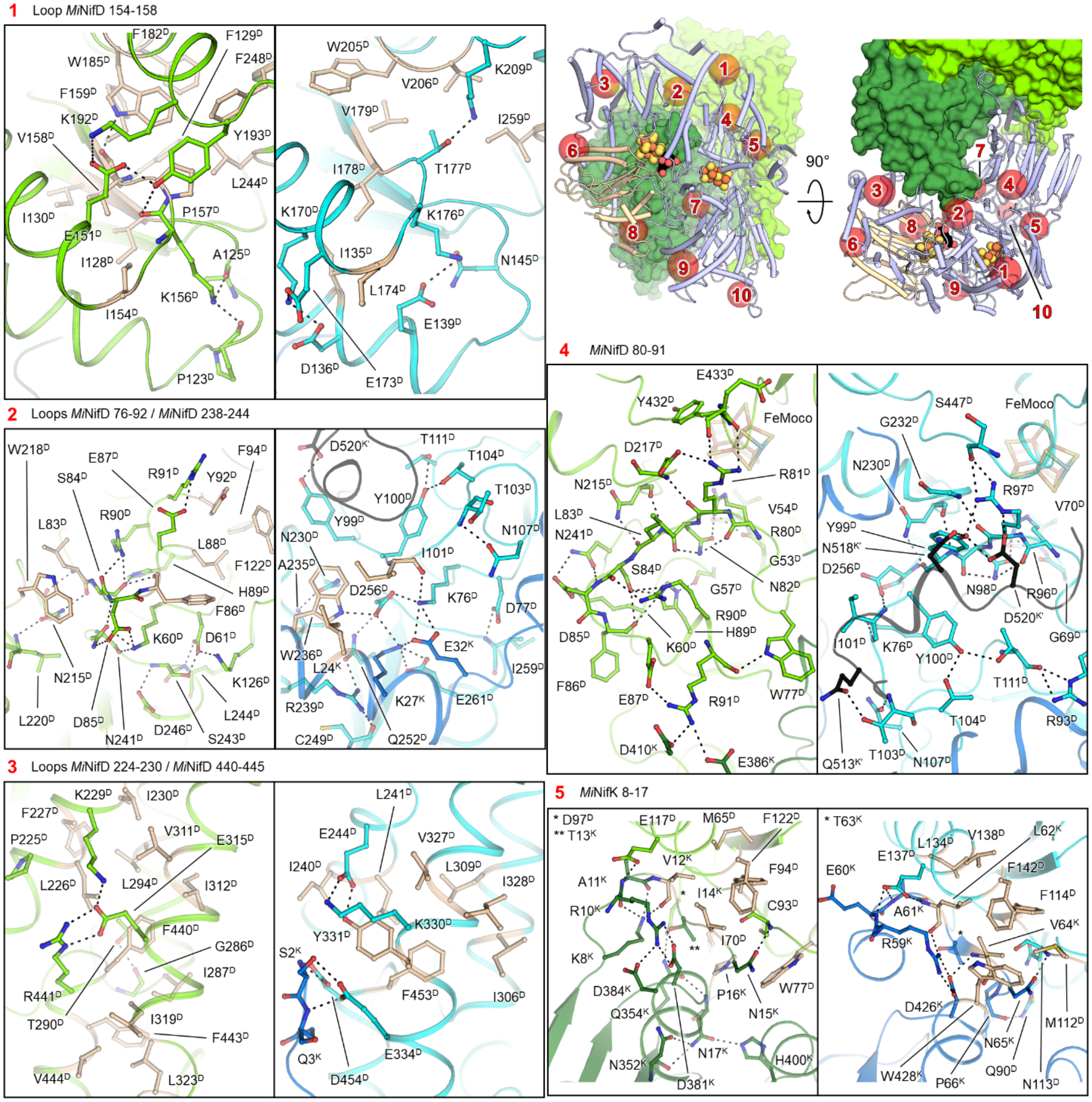

Supplementary Fig. 6. Hot spots in *Mi*NifDK. Top right panel presents the overview of *Mi*NifDK displayed in the same fashion as in Fig. 3e. The cartoon coloured in wheat indicates the αIII domain known for its intrinsic dynamics[2,3]. Local hot spots correspond to a structural reinforcement between *Mi*NifDK and *Av*NifDK through the apparition of salt bridges/hydrogen bonds (highlighted by dashes) or an increase in Van der Waals contacts through hydrophobic side chains (highlighted by wheat sticks). Inserts represent close-ups of the numbered area with the protein backbone shown in cartoon and the residue of interest in sticks or spheres. *Mi*NifD, *Mi*NifK, *Av*NifD, and *Av*NifK are colored green, dark green, cyan, and marine, respectively, in the close-ups. The opposite NifK dimer is colored in black. Prolines are also shown even if they do not contribute to the hydrophobic environment.

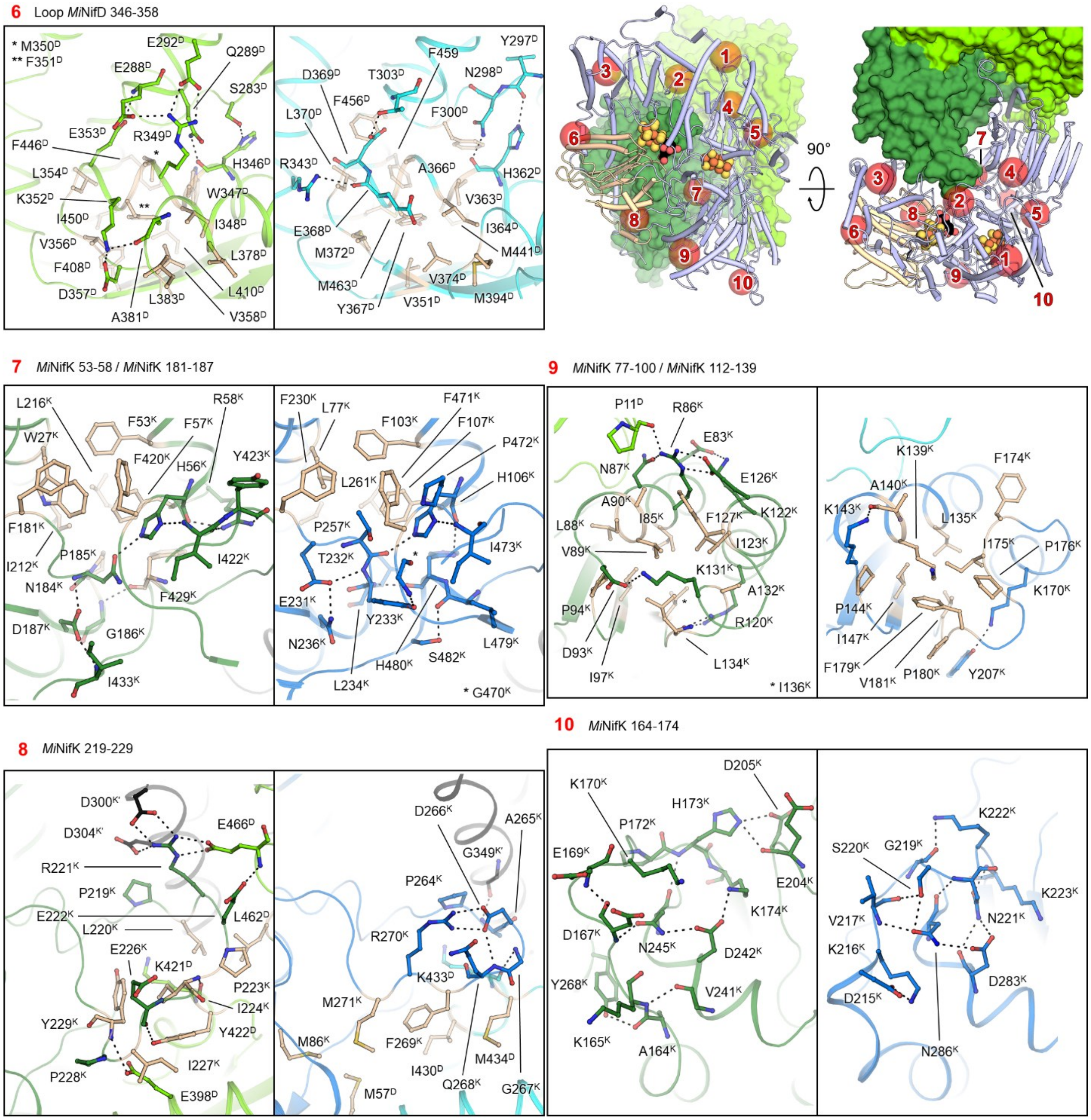

Supplementary Fig. 6 (continued). Hot spots in *Mi*NifDK. Top right panel presents the overview of *Mi*NifDK displayed in the same fashion as in Fig. 3e. The cartoon coloured in wheat indicates the αIII domain known for its intrinsic dynamics[2,3]. Local hot spots correspond to a structural reinforcement between *Mi*NifDK and *Av*NifDK through the apparition of salt bridges/hydrogen bonds (highlighted by dashes) or an increase in Van der Waals contacts through hydrophobic side chains (highlighted by wheat sticks). Inserts represent close-ups of the numbered area with the protein backbone shown in cartoon and the residue of interest in sticks or spheres. *Mi*NifD, *Mi*NifK, *Av*NifD, and *Av*NifK are colored green, dark green, cyan, and marine, respectively, in the close-ups. The opposite NifK dimer is colored in black. Prolines are also shown even if they do not contribute to the hydrophobic environment.

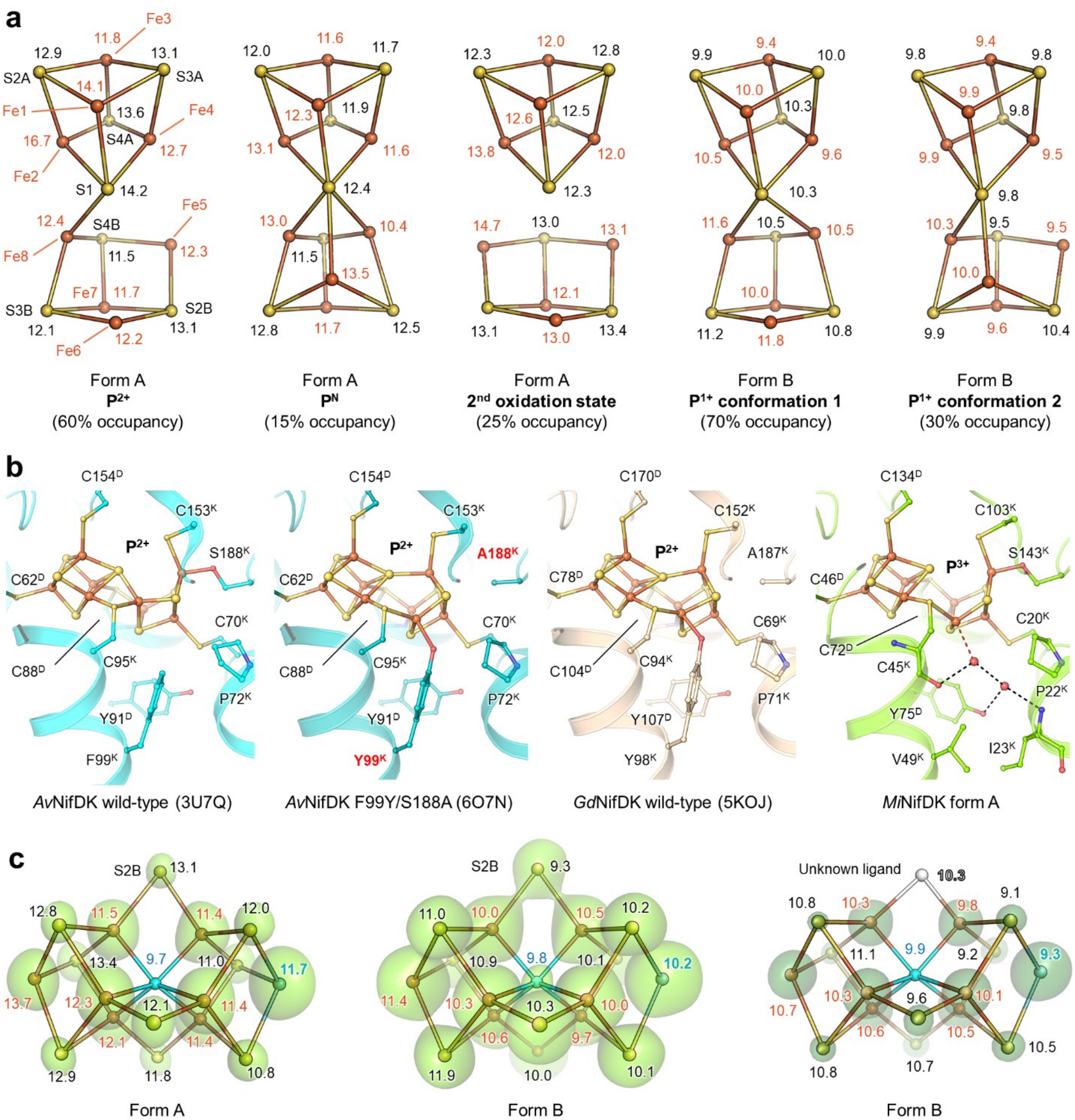

Supplementary Fig. 7. Comparison of the P-cluster binding in different models and omit maps for the FeMoco. **a**, B-factors of the P-cluster in the two forms. Omit maps for these P-clusters can be found in Supplementary Fig. 8. The different modelled states are displayed in balls and sticks, and individual atomic b-factors are labelled with S and Fe in black and orange, respectively. **b**, Differences in the oxidised P-cluster coordination between *Av*NifDK wild-type/variant, NifDK from *Gluconacetobacter diazotrophicus* (*Gd*NifDK) and *Mi*NifDK. Residues in the close vicinity of the P-cluster are displayed as balls and sticks, with waters in red spheres. Dashes show water´s interactions with the surroundings. The water-Fe8 distance for *Mi*NifDK shown in red is 2.7 Å. **c**, Omit maps and b-factors of the FeMoco in the two forms. $F_o - F_c$ maps are presented as a green transparent surface contoured to 15-σ, 6-σ, and 23-σ for form A, form B with modelled S2B, and form B with modelled ligand, respectively. The different modelled states are displayed in balls and sticks, and individual atomic b-factors are labelled with S and Fe in black and red, respectively. The unknown ligand was refined as a nitrogen.

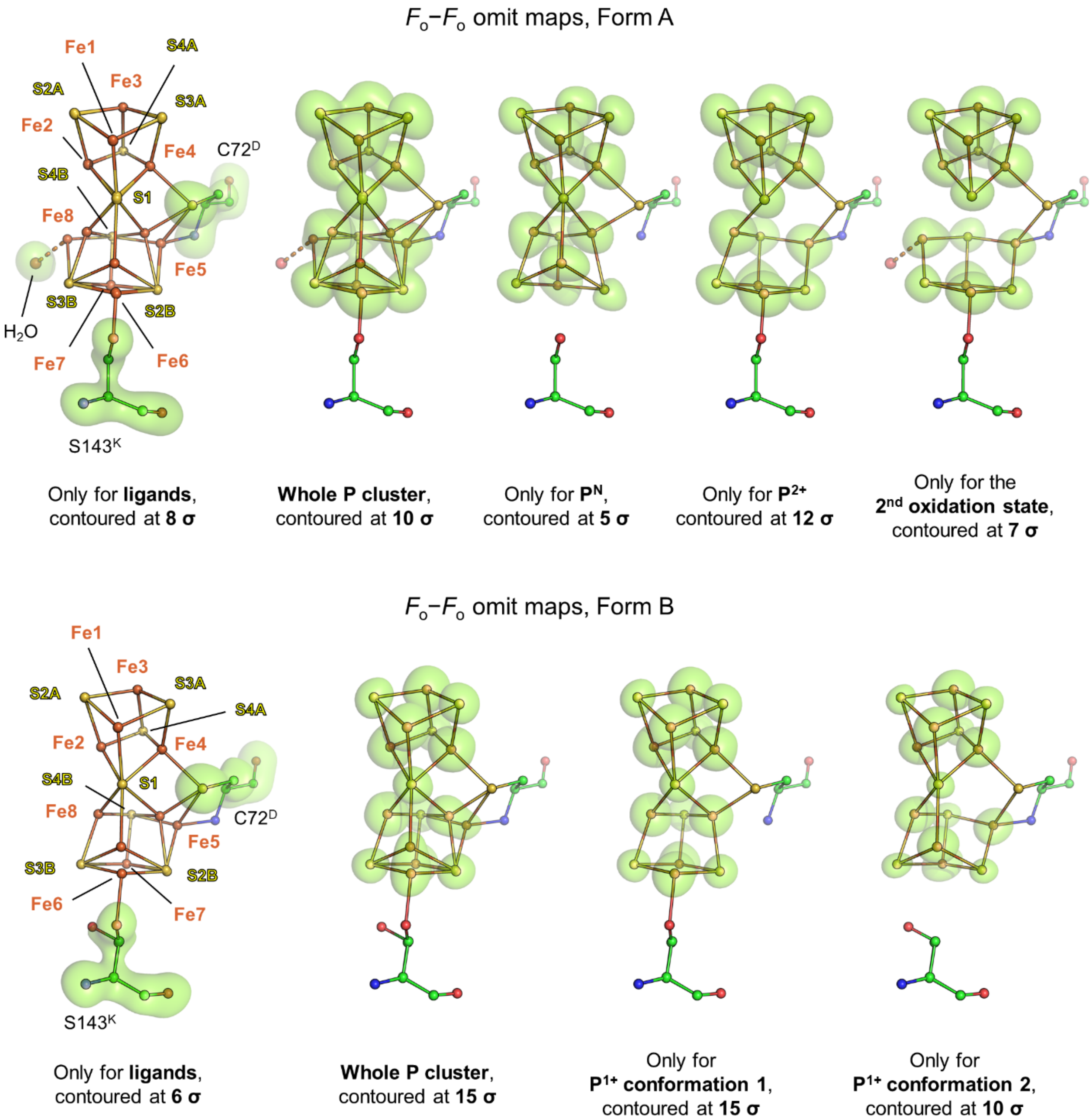

Supplementary Fig. 8. Omit map for all P-cluster states in forms A and B. P-clusters from each form in their different states are shown as balls and sticks with Fe and S colored orange and yellow, respectively. Cluster orientation is identical to that in Supplementary Fig. 7. F_o−F_c omit maps are displayed as transparent green surfaces.

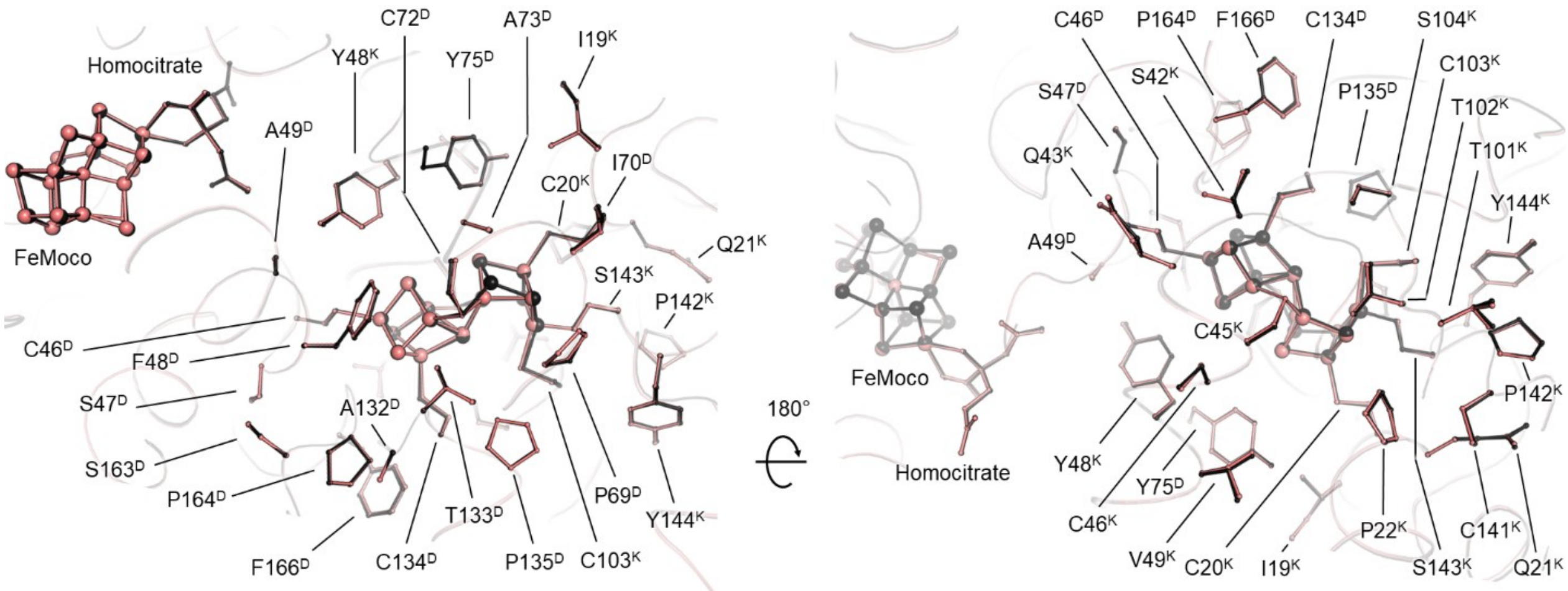

Supplementary Fig. 9. Superposition of form A and B, highlighting the spatial conservation of the residues. NifDK is shown as transparent cartoons, with residues in the first and second coordination sphere of the P-cluster displayed as sticks. FeMoco and P-cluster are represented as balls and sticks. Forms A and B are colored pink and black, respectively. The image is taken in the same pose as Fig. 4a.

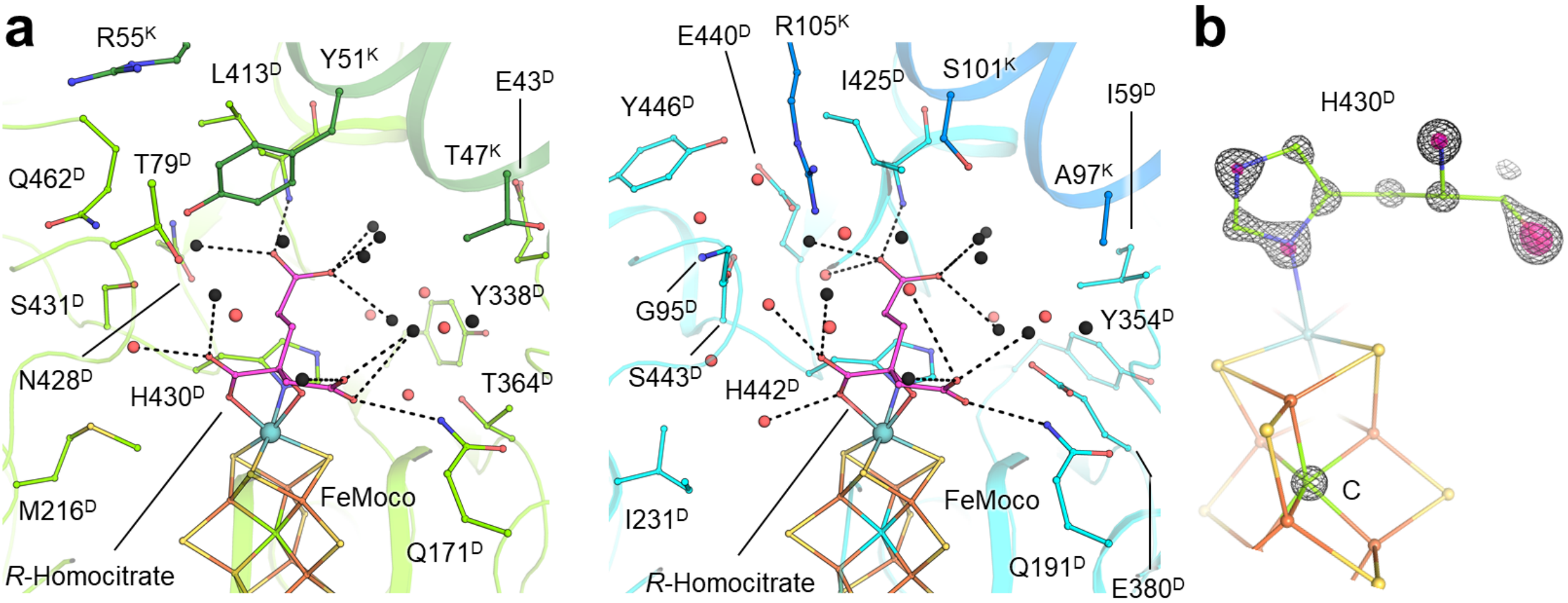

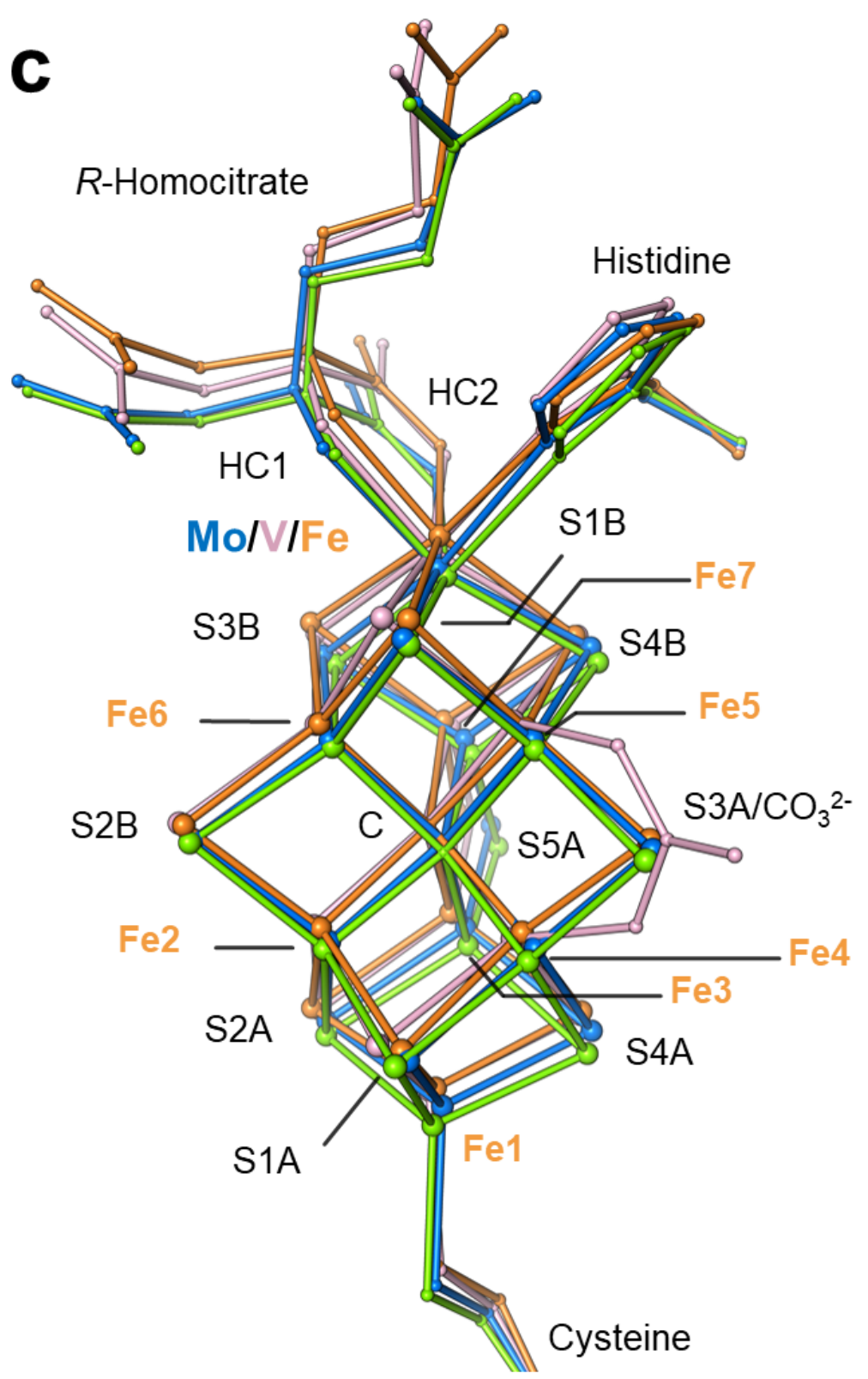

Bonds	Distances *Mi*Nif (Å)	Distances *Av*Nif (Å)	Atomic distances	Distances *Mi*Nif (Å)	Distances *Av*Nif (Å)
Fe1-Cys	2.16	2.27	S5A-Fe7	2.16	2.21
Fe1-S1A	2.28	2.31	S3A-Fe5	2.19	2.26
Fe1-S2A	2.29	2.27	C-Fe6	1.99	2.01
Fe1-S4A	2.27	2.29	C-Fe7	2.04	1.98
S2A-Fe2	2.27	2.25	C-Fe5	2.01	2.01
S2A-Fe3	2.26	2.27	Fe6-S3B	2.23	2.22
S1A-Fe2	2.27	2.26	Fe6-S1B	2.23	2.24
S1A-Fe4	2.26	2.29	Fe7-S3B	2.25	2.25
S4A-Fe3	2.27	2.25	Fe7-S4B	2.22	2.22
S4A-Fe4	2.27	2.29	Fe5-S1B	2.23	2.26
Fe2-S2B	2.22	2.20	Fe5-S4B	2.25	2.26
Fe2-C	2.00	2.01	S3B-Mo	2.36	2.37
Fe3-C	1.99	1.99	S1B-Mo	2.34	2.36
Fe3-S5A	2.20	2.23	S4B-Mo	2.35	2.35
Fe4-C	1.98	2.00	Mo-HC1	2.20	2.18
Fe4-S3A	2.21	2.24	Mo-HC2	2.10	2.21
S2B-Fe6	2.22	2.18	Mo-His	2.29	2.35

Supplementary Fig. 10. Comparison of the water network surrounding the homocitrate and comparison in the carbide-containing FeMoco. a. Water network in *Mi*NifDK (NifD light green and NifK dark green, left) and *Av*NifD (NifD cyan and NifK blue, right, PDB 3U7Q) around the *R*-homocitrate (pink). Waters are shown as spheres and coloured black or red if they collocate (with a distance below 0.7 Å) or not between *Mi*NifDK or *Av*NifDK. Residues in the close vicinity and ligands are depicted as balls and sticks. Dashes indicate direct contact between the homocitrate and the water molecules. **b**. Close-up of the FeMoco presenting an electron density for the interstitial carbon similar to a carbon rather than a nitrogen or oxygen. The $2F_o-F_c$ map is contoured at 4.5 σ (black mesh) and 6.5 σ (transparent pink surface). **c**. Superposition of the FeMoco, VFeco, and FeFeco, from *Mi*NifDK (green), *Av*NifDK (blue), *Av*VnfDK (pink), and *Av*AnfDK (orange). Superposition and distances were done on chain C.

References

1 Robert, X. & Gouet, P. Deciphering key features in protein structures with the new ENDscript server. Nucleic Acids Res 42, W320-324 (2014). https://doi.org/10.1093/nar/gku316

2 Warmack, R. A. & Rees, D. C. Structural evolution of nitrogenase states under alkaline turnover. Nat Commun 15, 10472 (2024). https://doi.org/10.1038/s41467-024-54713-0

3 Schmidt, F. V. et al. Structural insights into the iron nitrogenase complex. Nat Struct Mol Biol 31, 150-158 (2024). https://doi.org/10.1038/s41594-023-01124-2
