# Supplementary material for: Molecular basis of N2 fixation in a hyperthermophilic archaeon
Source: Nat Commun. 2026 Sep 8;17:9602. doi: 10.1038/s41467-026-77173-0 (PMC13554218; doi:10.1038/s41467-026-77173-0)
Supplement: Supplementary file 3 [file 41467_2026_77173_MOESM3_ESM.pdf]

nature portfolio

Peer Review File

Molecular basis of N_2 fixation in a hyperthermophilic archaeon

Corresponding Author: Dr Tristan Wagner

This file contains all reviewer reports in order by version, followed by all author rebuttals in order by version.

Attachments originally included by the reviewers as part of their assessment can be found at the end of this file.

Version 0:

Reviewer comments:

Reviewer #1

(Remarks to the Author)
The study of a hyperthermophilic archaean nitrogenase from Methanocaldococcus infernos is highly timely and of broad interest. Authors make three main claims:
1) exceptional thermostability and high activity,
2) a structurally simplified nitrogenase with features of all three known isoforms, and
3) trapping of distinct P-cluster and FeMo-cofactor states that unity mechanistic principles.

I recommend the publication of this manuscript in Nature Communications, pending thorough revision of the following points.

(1) Both models contains hydrogen atoms, which are generally not modeled in protein crystal structures.
(2) Model (PDB ID 9SPZ) contains unknown ligands assigned to chain H. These ligands have coordinates very similar to those of S2B atom in FeMoCo.
(3) In model (PDB ID 9SPZ), one sulfide is located unreasonably close to Gln171 in NifD. Please reconsider this assignment.
(4) Model (PDB ID 9SPZ) include two FeMoCo, each with occupancy set to 50%. Please remove the one and set 100% of occupancy for the other.
(5) Model (PDB ID 9SPZ) has two P-cluster-like ligands named PCL and PCM. For reference, the reduced P-cluster has been deposited as CLF, and the oxidized form as 1CL.
(6) The occupancy of P-clusters in Model (PDB ID 9SQ3) is too low. Moreover, the electron density corresponding to the two proposed P-cluster types is unclear. Although the authors have also assigned two 4Fe-4S clusters at this position, these too lack well-defined density.
(7) If the authors want to claim the oxidation states of P-cluster, an accompanying EPR spectrum is required for confirmation.

Reviewer #2

(Remarks to the Author)
This manuscript by Maslać et al. describes the first structural characterization of the catalytic component (NifDK) of the nitrogenase enzyme from a thermophilic organism, Methanocaldococcus infernus. This characterization of MiNifDK nitrogenase is quite interesting for a multitude of reasons as the authors specified, especially the fact that this nitrogenase likely predates the most commonly studied versions and represents a minimal nitrogenase MoFeP. The authors provide high resolution crystal structures of the catalytic component, MiNifDK, that demonstrate different conformers of both the P-cluster and FeMmoco that are attributed to different oxidation states. These structures are indeed noteworthy and original.
While these findings are of high interest to the nitrogenase community, I think that there are important concerns that must be addressed. Thus, I think the manuscript should be re-submitted for review after major revisions. (1) Most importantly, there are key experiments and analyses are missing that are needed to support their conclusions. (2) There are also studies mentioned in the manuscript that are left uncited, and (3) some figures are very unclear for the reader.
Missing experiments, analyses, and discussion:
• Conclusive identification of NifI1 and NifI2 - In the paragraph beginning at line 90, the authors identify SDS-PAGE bands as NifI1 and NifI2. However, these proteins are only identified based on migration in the gel. In order to conclusively identify

them, further experiments need to be carried out. Either a western blot (assuming antibodies are available) or in-gel digestion coupled with mass spec would suffice.
Additionally, In Fig 1d, the authors state that the 2-oxoglutarate treatment releases these proteins from MiNifDK before the final purification steps. However, there are still two bands that between 10-15kDa in size in the "Purified NifDK" lanes, they are just shifted slightly relative to before the treatment. Identification of these bands is crucial to corroborate the conclusions that they drew.
• Comparison of the residues around the P-cluster and in the ET relay chain between the P-cluster and FeMoco – Line 186 states, "Residues in the surroundings of the P-cluster, including the electron relay, are perfectly superposable between both forms…". An extended data table with RMSD values of just these residues should be included to support the claim that they are "perfectly superposable".
• Omit maps of the P-clusters – I find the identification of the percent occupancy of different states of the P-clusters in the crystals very interesting. This would be even more convincing with proper omit maps as extended data figures, which I would expect to show elongation/multiple positions around relevant atoms/residues.
• Spectroscopy to confirm electronic states of the P-cluster in the as-purified samples – The authors postulate that MiNifDK can actually accommodate a stable P3+ oxidation state of the P-cluster. I am left wondering- is this reversible? Within the nitrogenase literature, there have been studies observing the P3+ state in the MoFeP and FeFeP from other organisms. However, oxidation of the P2+ state to P3+ is typically considered irreversible.
Additionally, the authors suggest multiple explanations as to why the two crystals exhibited different P-cluster occupancies, with the most plausible reason being the timeframe difference in crystallization. This suggests that a sample of protein left in the glovebox left for a month would be more oxidized than one freshly purified (from oxygen contamination?).
EPR experiments could be carried out to demonstrate (1) whether the P3+ is reached at all, and if so, whether it is reversible, and (2) if the P3+ state in the crystal structure actually arose from oxidation within the glove box.
However, I would understand if EPR experiments are beyond the scope of this paper. If the authors choose not to do EPR spectroscopy, then I think it would be pertinent to add a few sentences about what is already known about the P3+ state and that future spectroscopic experiments could be carried out to test their hypotheses. Also, as the authors mentioned, it is very interesting that they were able to capture what appears to be the P1+ state of the P-cluster without special crystallization conditions to carefully regulate the reduction potential of the crystallization solution. While they do provide some hypotheses for why this happened, discussion related to reduction potentials should be included. Additionally, if it is easier to isolate the P1+ state in Mi than in other nitrogenases, this could also be corroborated with spectroscopy.
• Turnover state in the presence of O2 without FeP? -The authors clearly hypothesize that their "form B" or "turnover state" may have arisen from "crystallization delay… uncontrolled oxidation event (e.g., partial O2 contamination in the glove box) …" but then claim that the form B's FeMoco is in a "turnover state". In the discussion, (lines 266-267) they state "...as well as the rapid purification and crystallization under strict exclusion of O2 and dithionite…" as why form B exists. These statements are directly contradictory, and I am not convinced of a "turnover state" when the authors themselves have doubts about the oxygen exclusion of their glove box.
I do think the comparison to other turnover states is interesting. It might be in a state resembling an intermediate- I just don't understand how that could happen with (1) oxygen in the glovebox and (2) no reductant of MoFeP (ie no FeP). The authors would need to show experiments demonstrating this ability of the nitrogenase from this organism to reduce substrates (such as N2) in the presence of DTT without FeP for this to be a convincing sound hypothesis.

Missing key citations The following pieces of information lack vital citations:
• Line 59 "…Azotobacter vinelandii, whose disproportionately large set of nif genes…" - doi: 10.1128/jb.171.2.1017-1027.1989
• Line 104 "…the maximal temperature allowed by the ATP-regeneration system…"- provide a citation for this (I don't know of one) or provide an SI figure showing data to support this
• Line 119 "… canonical NifD2K2 assembly in which dimerization occurs via NifK…" – cite https://doi.org/10.1021/bi00079a006
• Line 151-152 "… known for its high intrinsic flexibility and participating in FeMoco delivery and catalysis" – should also cite DOI: 10.1126/science.abq7641
• Lines 176-177- "… was proven by exchanging the air of Phe99K/Ser188K to Tyr99K/Ala188K in AvNifDK."- cite https://doi.org/10.1021/jacs.9b04555
• Line 213- "… corroborating that the FeMoco is chemically identical to that of AvNif…" this should read "corroborating that the FeMoco is chemical identical to that of Av NifDK…" and should cite the following 2 papers that identified the central atom of FeMoco to be a carbide: DOI: 10.1126/science.1214025 and DOI: 10.1126/science.1206445

Figures The following figures need modifications:
• About a quarter of the intro focuses on the clusters and ET. The reader must understand this to understand the results and discussions later in the manuscript. I think it would be highly beneficial to move extended data fig 1a to the main text for this reason.
• Figure 1- the legends in 1a and 1b are swapped. Protein bands in 1d should be labeled similar to 1c. It looks like there is still NifI, even when it is supposed to be removed.
• Figure 2- It is very hard to see the "transparent white" portion.
• Figure 3- The transparency/depth cuing on this figure seems off. It is hard to discern what is important and where residues are in relation to the clusters. Also, making the labels of the residues match in transparency to the depiction of them in the model would be more clear than having all labels at 100% opacity. Also, indicating the ET chain would be helpful.
• Figure 4- I am fairly certain that the models shown are the structures from this paper and not from the PDB, but it is unclear in the caption and main text. "1st and 2nd spheres" should say primary and secondary coordination spheres. I also think calling the structure a turnover structure could be misleading.

• Extended figure 1b- the colors in the plot don't match the legend. Added N2 in mmol isn't a very useful measure. Could this be converted to concentration (partial pressures)? Also, it should be mentioned in the caption that "as isolated" is DTT reduced.

Other comments that need to be addressed:
• When the amount of N2 used during growths is mentioned throughout the paper (main text and figs), it says the amount in mmol. Putting this in terms of concentration such as partial pressures makes more sense.
• Line 110 could be further clarified by adding to the end: "… stalling the enzyme at lower temperatures.
• The central atom in the P-cluster is called a "sulfur" throughout the manuscript, but it should be "sulfide".
• For the paragraph beginning at line 164, it would be beneficial to add an intro sentence or two that clearly and concisely introduces the known oxidation states of the P-cluster and their corresponding conformational changes, with appropriate citations. As it is currently written, it is scattered throughout the paragraph and isn't clear to readers who might not be intimately familiar with the P-cluster. Rather than citing a review, cite https://doi.org/10.1021/bi9626665 and https://doi.org/10.1074/jbc.RA118.002435 (citation #57 in this manuscript)
• Line 165 should be clarified with "This 2e– oxidized state…"
• The authors say that they don't use diothionite during purification and crystallization because it could possibly be a "non-innocent reductant for metalloproteins". However, they still use it in their activity assays as the sacrificial electron donor. To me, their reason for not using it thus isn't justified, and they need to clarify in the main text that they still used it in the assays, and an explanation as to why it is ok to use in assays but not other parts of their methods (which I don't understand).
• In the author's previous paper (Maslać et al. 2022 Comparative Transcriptomics Sheds Light on Remodeling of Gene Expression during Diazotrophy in the Thermophilic Methanogen Methanothermococcus thermolithotrophicus), the authors state "a supplementation with the concentration of 100 uM [W] gave unreproducible results". In this manuscript, the authors use 100uM, citing the aforementioned paper. Can the authors please explain why they changed growth media to one that previously gave unreproducible results?
Best,
Hannah Rutledge

Reviewer #3

(Remarks to the Author)
In the present study, Maslać et al. describe the novel features of MiNifDK, the FeMoco nitrogenase from a hyperthermophilic archaeon. The authors successfully isolated $MiNifD_2K_2$ from an organism grown at 75 °C (its natural habitat is 93 °C) and demonstrated that it exhibits exceptional thermostability. This significantly surpasses that of previously characterized homologues and is accompanied by the highest N_2-reduction activity at the highest temperature reported to date. Using two complementary purification and crystallisation strategies, the authors captured structurally distinct states of the metal clusters in MiNifDK. Four distinct redox states of the P-cluster, which had previously been identified in nitrogenase homologues, were observed. Additionally, two states of the FeMoco active site were resolved: a resting state and a turnover state. Notably, the turnover state had not previously been identified in FeMoco nitrogenase, representing a significant advance in the field. These findings provide important mechanistic insights into nitrogenase function and are expected to inform future efforts to develop and improve the ammonia production process.
Overall, the manuscript is well written, carefully interpreted, and clearly presented. The reviewer strongly recommends this manuscript for publication in Nature Communications.

However, the following minor comments must be addressed.

Minor comments

Line 38: How about providing a brief overview of the redox states of the P-clusters and the FeMoco site of nitrogenase, the two main observations in this report?

Line 51-55: For the general readers, it would be helpful to include a more detailed structural description of the P-cluster, FeMoco, FeV, and FeFe cofactors, including their compositions such as MonFenSn…, in the main text.

Line 76: It seems that Figures 1a and 1b have been mixed up. Please check the figures.

Line 79: Why is mol mass (mmol) used in the text rather than concentration (mM) used in the figures? For easy comparison, please use only one unit. For example, change the unit in the figures to molar mass.

Line 125: If possible, provide an X-ray fluorescence edge scan of the Mo and Fe, and indicate the energy at which anomalous data were collected in an extended data figure.

Line 161: Define the superscripts K and D when they are first used in the text.

Line 181: Clearly describe conformations 1 and 2 of the P1+ state in the text. Change 'the P1+ state in which' to 'the P1+ state (70% occupancy of conformation 1) in which'.

Line 184: Change 'occupancy of 30%' to 'occupancy of 30% (Conformation 2)'.

Line 185: Indicate new bonds for the P1+-conformation 2 (Fe5 to the amide backbone of Cys72D and Fe6 to the central S) by arrow or star on the fig. b, as these are difficult to identify at first glance.

Line 210: Indicate the H-bonding network in Fig. 4a, for example using a dashed bond.

Line 479-486: Please incorporate this paragraph into the legend of Fig. 2a or the main text where appropriate, as it is not very convincing and it is difficult to understand the type of maps presented and how they are made. If this is not feasible, please refer to the "Methods" section for a detailed explanation of how the maps are calculated and what they are.
Line 481: change "A difference maps" to A anomalous difference Fourrier maps".

Line 529: In Fig. 1b, comment on why the culture exhibits a longer lag phase in the presence of 100 µM Na_2MoO_4.

Line 543: 1) Change 'Anomalous difference maps' to 'Anomalous difference Fourier maps'.
2) Are these maps calculated from the high-energy side, or are the double difference maps calculated from the high-energy side minus the low-energy side? If so, please mention in the legend text how the map was calculated from the low-energy side.
3) In the figure legend, mention the energy at which the anomalous difference Fourier maps were calculated on the high-energy side (xx Å) or the low-energy side (yy Å) of the metal absorption edge.

Line 545: in the right graph of Fig. 2a, what does 'overall' mean? It seems that the authors indicated it as 'NifD2K2'. If this is the case, change 'overall' to 'NifD2K2'.

Line 548: in the left panel of fig. 2c,
1) There are 11 red spheres representing the hot spots, but only 10 are shown in the extended data Fig. 7. What about the 11th sphere? Would it be from other subunit. Please comment on this.
2) The reviewer does not see the benefit of superposing MiNifDK and AvNifDK, or of showing the absence of metal clusters, in the figure. Since the comparison of the two structures for the hot spots is clearly shown in the extended data Fig. 7, it is not necessary to show the superposition again here, as this actually makes the view more complex. The reviewer thinks that the structure in the top right panel of the extended data Fig. 7 is more informative and would fit better in the main figure.
Line 550: in the fig. 2c right graphs for the number of hydrogen bonds/salt bridges, what stands for dark and light green bars for?

Line 572: In Fig. 4a, 'Q462K´' is written, but there is no explanation of the " ´ " in the legend.

Line 583: In Extended Data Table 1,
1) the Form A crystal shows an increase in ⟨I/σ(I)⟩ with increasing X-ray energy, as expected. However, for the two anomalous Mo data sets collected at 19.9 and 20.1 keV, ⟨I/σ(I)⟩ decreases as the data-collection energy increases. In addition, higher-energy data collection is generally expected to allow access to higher-resolution diffraction, yet this is not observed for the Form B crystal.
2) The number of unique reflections can be approximately doubled when anomalous data are processed with Friedel mates treated as independent reflections, rather than being merged. However, for the datasets presented here, the number of reflections between the anomalous data and the high-resolution data does not match this expectation.
Please comment on these observations. Additionally, indicate each data set how you treated the Friedel mates for data processing.

Line 620: provide a concentration of the as-isolated MiNifDK in the legend for comparison with the added DT.

Line 689: in Extended data fig. 9a,
1) All the residues indicated are from the NifD subunit (D superscript), particularly Q462D. However, Q462 is from the NifK subunit in Fig. 4a. Check for inconsistencies and correct them.
2) There are two asterisks ('*') in H430D/H442D and a His residue in both figures of Fig. 9a. However, there is no explanation in the legend about the meaning of these.

Version 1:

Reviewer comments:

Reviewer #1

(Remarks to the Author)
I have carefully reviewed the authors' comprehensive rebuttal and the revised manuscript. The authors have diligently and logically addressed all my concerns, providing sufficient structural evidence and making appropriate textual modifications. I recommend the manuscript for publication in its current form.

Reviewer #2

(Remarks to the Author)

Comments are attached as pdf.

Reviewer #3

(Remarks to the Author)
The authors have thoroughly addressed all of my questions and concerns. I am satisfied with the revisions and believe that the manuscript is now suitable for publication in Nature Communications. I recommend acceptance for publication.

Open Access This Peer Review File is licensed under a Creative Commons Attribution 4.0 International License, which permits use, sharing, adaptation, distribution and reproduction in any medium or format, as long as you give appropriate credit to the original author(s) and the source, provide a link to the Creative Commons license, and indicate if changes were made.
In cases where reviewers are anonymous, credit should be given to 'Anonymous Referee' and the source.
The images or other third party material in this Peer Review File are included in the article’s Creative Commons license, unless indicated otherwise in a credit line to the material. If material is not included in the article’s Creative Commons license and your intended use is not permitted by statutory regulation or exceeds the permitted use, you will need to obtain permission directly from the copyright holder.
To view a copy of this license, visit https://creativecommons.org/licenses/by/4.0/

We would like to thank all reviewers for their time and consideration of our manuscript. We answered all points to the best of our ability and revised the manuscript according to their comments.

Reviewer #1:

(1) Both models contains hydrogen atoms, which are generally not modeled in protein crystal structures.

(Our response) Hydrogens were intentionally retained in both deposited structures because they significantly affect the final R-factor and R-free. Form A at 1.37 Å has a Rfactor/Rfree of 12.33/15.56 % with modelled hydrogens and 13.40/16.35 % without hydrogens. Form B at 1.21 Å has a Rfactor/Rfree of 12.08/14.60 % with modelled hydrogens and 13.27/16.56 % without hydrogens. It attests that in the present case, modelling hydrogen is beneficial to the agreement between the crystallographic model and the experimental X-ray diffraction data, resulting in higher quality maps.
A sentence has been added in the Materials and Methods on that regard.
Lines 493-497: "It must be noted that models were deposited with hydrogens, as they significantly affect the final R-factor and R-free. Form A at 1.37 Å has a Rfactor/Rfree of 12.33/15.56 % with modelled hydrogens and 13.40/16.35 % without hydrogens. Form B at 1.21 Å has a Rfactor/Rfree of 12.08/14.60 % with modelled hydrogens and 13.27/16.56 % without hydrogens."

(2) Model (PDB ID 9SPZ) contains unknown ligands assigned to chain H. These ligands have coordinates very similar to those of S2B atom in FeMoCo.

We downloaded the .pdb and .mtz files processed automatically by Nature Communications and observed discrepancies with the files obtained from the European Protein Data Bank (PDB). We are unsure where the issues are coming from, but the chain assignment does not represent the ones deposited in both models. To fix this problem, we have now uploaded the .cif and .pdb files of the processed model obtained from the PDB. In PDB ID 9SPZ, the only ligand in chain H is HCA, which is the homocitrate coordinating the FeMo-cofactor (FeMoco). The ligand is correct.
However, in PDB ID 9SQ3, we indeed have an unknown ligand bound to the FeMoco. This unknown ligand has been a matter of debate (modelled as a nitrogen in the VFe-nitrogenase and as an oxygen in the FeFe-nitrogenase). Experts in the field (Prof. Einsle and Dr. Björnsson) advised us to model it as an unknown ligand.

(3) In model (PDB ID 9SPZ), one sulfide is located unreasonably close to Gln171 in NifD. Please reconsider this assignment.

In PDB ID 9SPZ (form A), there is no sulfide located close by Gln171 in NifD (S2B is still at a reasonable distance). However, in PDB ID 9SQ3, containing the two different conformations at the FeMo-co site, there is indeed a sulfide clashing with Gln171. However, we modelled the sulfide and the Gln171 in a double conformation as follows: in conformer A, corresponding to the "resting state", S2B is bound to the FeMoco and the side chain of Gln171 points towards Gly45 in NifD (no clashes). In conformer B, corresponding to the "alternate state", S2B migrates in front of the Gly45. It is not present on the FeMoco and is replaced by an unknown

ligand. The Gln171 side chain switches conformation and is in front of the unknown ligand. In these alternative conformations, the modelled sulfides do not clash.

(4) Model (PDB ID 9SPZ) include two FeMoCo, each with occupancy set to 50%. Please remove the one and set 100% of occupancy for the other.

Model PDB ID 9SPZ includes a single FeMoco per active site at 100% occupancy.
However, in PDB ID 9SQ3, a mixture of states is observable for the FeMoco: a fully loaded state with S2B and a loaded state with the unknown ligand. Based on the electron density from the near-atomic resolution maps, advice from peers, and from the European PDB, we modelled both states with an occupancy of 50/50. Modelling a single FeMoco at 100% occupancy won´t reflect the mixture of two states and would generate a clash with the alternative conformer of Gln171. For this reason, we prefer to keep both FeMoco 50/50 alternative conformations, in which conformer B, containing the bound unknown ligand, does not have S2B.

(5) Model (PDB ID 9SPZ) has two P-cluster-like ligands named PCL and PCM. For reference, the reduced P-cluster has been deposited as CLF, and the oxidized form as 1CL.

In PDB ID 9SPZ, the European PDB assigned the three modelled P-clusters with the following ids:
Reduced P-cluster (P^N): CLF
Oxidized P-cluster (P^{2+}): 1CL
Second oxidised state: F4S+SF4
The usual nomenclature is therefore respected.
In PDB ID 9SQ3, the European PDB created new ligands to fit the two alternative conformers of the P-clusters in the P^{1+} state: A1JPW and A1JPX.
The correct ligand nomenclature can be observed in the newly uploaded .cif and .pdb models.

(6) The occupancy of P-clusters in Model (PDB ID 9SQ3) is too low. Moreover, the electron density corresponding to the two proposed P-cluster types is unclear. Although the authors have also assigned two 4Fe-4S clusters at this position, these too lack well-defined density.

We are surprised by this comment. The electron density ($2F_o{-}F_c$) is extremely clear for all P-clusters in PDB ID 9SQ3 as shown in the following snapshot:

Electron density of the P-cluster sites in PDB 9SQ3

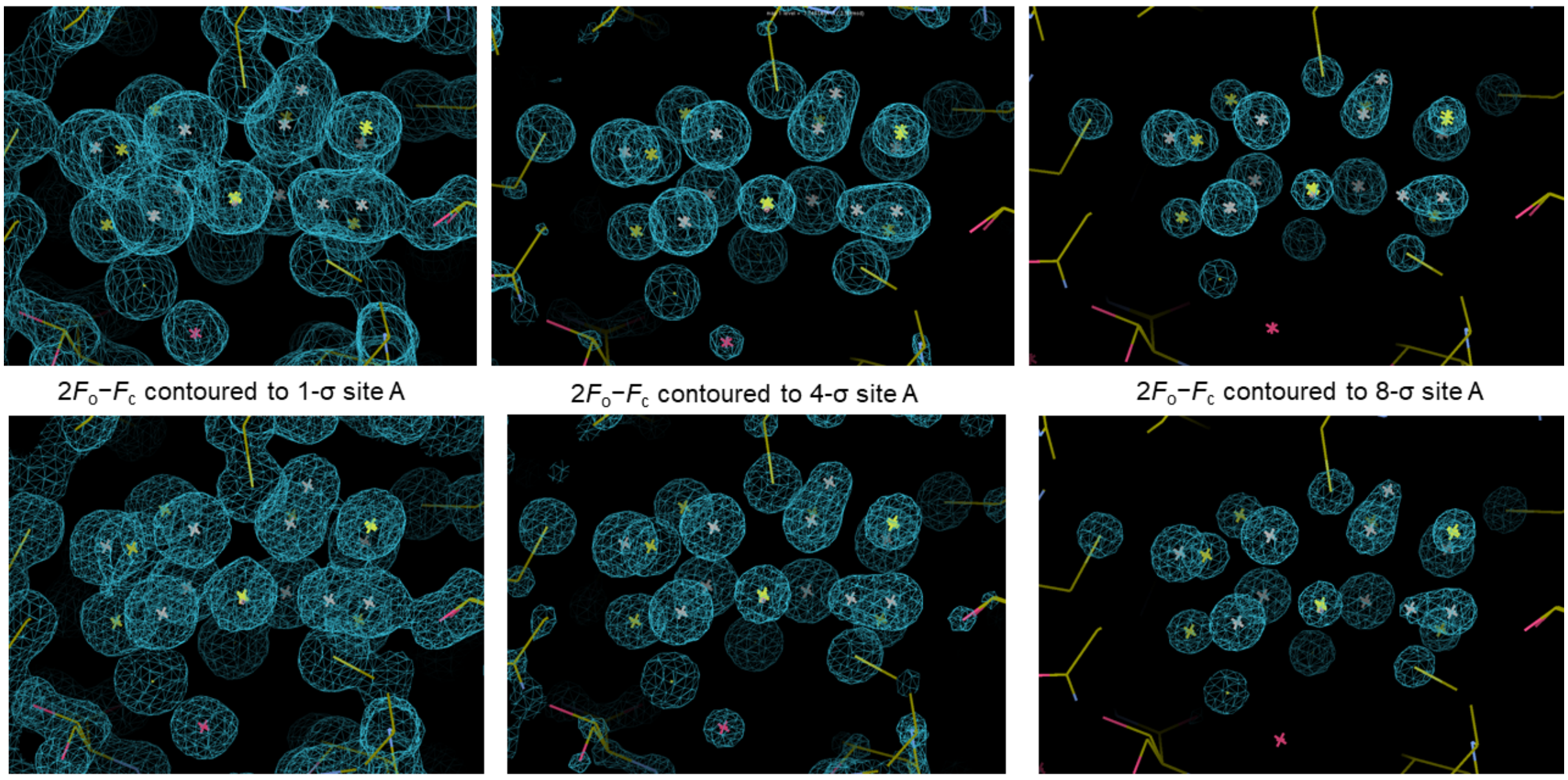

$2F_o-F_c$ contoured to 1-σ site A | $2F_o-F_c$ contoured to 4-σ site A | $2F_o-F_c$ contoured to 8-σ site A

$2F_o-F_c$ contoured to 1-σ site B | $2F_o-F_c$ contoured to 4-σ site B | $2F_o-F_c$ contoured to 8-σ site B

The modelled metalloclusters in PDB ID 9SQ3 are two alternative conformers of the P-cluster in the P^{1+} states. The accuracy of the modelled conformers and their occupancies has been assigned based on the difference map after refinement (materials and methods), b-factors (new Extended Data Fig. 7), and confirmed with omit maps (new Fig. 4b).
The same is true for PDB ID 9SPZ, as shown in new Fig. 4b and new Extended Data Fig 7. In both models, the omit map shows a clear presence of the P-clusters. The two clusters ([4Fe-4S] and [4Fe-3S]) modelled in 9SPZ are the result of the second oxidised state. The combination of ligands (SF4 + F4S) has been recommended by the European PDB.

We now present a supplementary Figure containing an omit map for all modelled P-clusters (Supplementary Fig. 1).

(7) If the authors want to claim the oxidation states of P-cluster, an accompanying EPR spectrum is required for confirmation.

We acknowledged the reviewer's argument. While EPR spectroscopy experiments are beyond the scope of the present study, we have clarified throughout the text that our observations could be corroborated by future spectroscopic experiments (please see lines 192-193, and 249-250 in the revised manuscript). Instead of using the term P^{3+}, we now refer to the observed state as "the second oxidised state".

Reviewer #2:

(1) Conclusive identification of NifI1 and NifI2 - In the paragraph beginning at line 90, the authors identify SDS-PAGE bands as NifI1 and NifI2. However, these proteins are only identified based on migration in the gel. In order to conclusively identify them, further

experiments need to be carried out. Either a western blot (assuming antibodies are available) or in-gel digestion coupled with mass spec would suffice.

The reviewer is correct, we did not confirm $NifI_{1,2}$ identity at the stage of manuscript submission. We were biased because we had already identified these regulators, which were later published as a preprint from Maslać N, Bolte P. Müller M-C and Wagner T: https://doi.org/10.64898/2025.12.07.692841

We now cite the preprint in the text with an explanation sentence:

Lines 104-105 "The identification and structural characterisation of *Mi*$NifI_{1,2}$ alone or in complex with *Mi*NifDK have been recently reported (please see reference 49 in the revised manuscript, https://doi.org/10.64898/2025.12.07.692841)."

(2) Additionally, In Fig 1d, the authors state that the 2-oxoglutarate treatment releases these proteins from MiNifDK before the final purification steps. However, there are still two bands that between 10-15kDa in size in the "Purified NifDK" lanes, they are just shifted slightly relative to before the treatment. Identification of these bands is crucial to corroborate the conclusions that they drew.

As mentioned in the previous point, the second manuscript presents another preparation of purified $NifDKI_{1,2}$ and NifDK (Supplementary Fig. 1b in the BioRxiv preprint by Maslać N et al.), in which additional bands are not observed in purified NifDK, suggesting that they are contaminants. The overall work in the preprint confirms our conclusion that the addition of 2-OG and MgATP disrupts the *Mi*NifDK–*Mi*$NifI_{1,2}$ complex, consistent with previous reports in the literature (e.g., https://doi.org/10.1073/pnas.0602278103).

(3) Comparison of the residues around the P-cluster and in the ET relay chain between the P-cluster and FeMoco – Line 186 states, "Residues in the surroundings of the P-cluster, including the electron relay, are perfectly superposable between both forms...". An extended data table with RMSD values of just these residues should be included to support the claim that they are "perfectly superposable".

This is a very fair point from the reviewer that we should not overstate the claim using an adjective such as "perfectly". We wanted to highlight the striking spatial conservation of these residues and provide a supplementary Figure of the structural alignment. Since "perfectly" could be interpreted as a rmsd of 0 Å, we removed the term (line 200) and simply cite the Supplementary Figure 2.

(4) Omit maps of the P-clusters – I find the identification of the percent occupancy of different states of the P-clusters in the crystals very interesting. This would be even more convincing with proper omit maps as extended data figures, which I would expect to show elongation/multiple positions around relevant atoms/residues.

Based on the reviewer´s comment, we have now removed the omit map from the new Extended Data Fig. 7 and created a new supplementary figure (Supplementary Figure 1) with omit maps for all states of the P-clusters reported in this study.

(5) Spectroscopy to confirm electronic states of the P-cluster in the as-purified samples – The authors postulate that MiNifDK can actually accommodate a stable P^{3+} oxidation state of

the P-cluster. I am left wondering- is this reversible? Within the nitrogenase literature, there have been studies observing the P3+ state in the MoFeP and FeFeP from other organisms. However, oxidation of the P2+ state to P3+ is typically considered irreversible. Additionally, the authors suggest multiple explanations as to why the two crystals exhibited different P-cluster occupancies, with the most plausible reason being the timeframe difference in crystallization. This suggests that a sample of protein left in the glovebox left for a month would be more oxidized than one freshly purified (from oxygen contamination?)

We thank the reviewer for this point. The mixture of oxidised states has been derived solely from electron-density interpretation and the remaining F_o–F_c signals after refinement. The main difference between the two forms is the duration of crystal formation and fishing. The kinetics of crystal apparition might have been influenced by protein concentration, as hypothesised in the text. Since the cells and protein sample were treated in a very similar fashion, with O_2 at < 5 ppm, we did not anticipate oxidation during the purification procedure. However, the glovebox used to store crystals can be sporadically exposed to O_2 during harvesting when liquid nitrogen is transferred inside (with O_2 ppm rising to 30 - 60). This might have oxidised the NifDK crystal of form A over the long storage period.

Regarding reversibility, most of the literature we found indicates that it is not reversible (now added on lines 59-60). In our case, the Fe8 is in contact with a water molecule, which is less stable than the tyrosine interaction in *Gluconacetobacter diazotrophicus* NifDK. Therefore, we agree that the Fe might be slowly labile. However, we did not want to state anywhere in the manuscript that the observed P^{3+} state could be reversible. As mentioned later, we soften our message and use the term "second oxidised state" instead of the previously P^{3+}.

Lines 189-193: "Based on the electron density profile and structure refinement, we propose that this state could imply a further oxidation of the P-cluster in a P^{3+}, and we suspect its preservation to be due to the high enzyme stability caging the oxidised cluster and preventing its collapse. This hypothesis could be further tested using spectroscopic approaches (e.g., electron paramagnetic resonance experiments), which are beyond the scope of the present study."

The repertoire of P-cluster redox states obtained by soaking crystals in oxidants and reductants is beyond the scope of this work and will be addressed in a future study.

(6) EPR experiments could be carried out to demonstrate (1) whether the P3+ is reached at all, and if so, whether it is reversible, and (2) if the P3+ state in the crystal structure actually arose from oxidation within the glove box. However, I would understand if EPR experiments are beyond the scope of this paper. If the authors choose not to do EPR spectroscopy, then I think it would be pertinent to add a few sentences about what is already known about the P3+ state and that future spectroscopic experiments could be carried out to test their hypotheses. Also, as the authors mentioned, it is very interesting that they were able to capture what appears to be the P1+ state of the P-cluster without special crystallization conditions to carefully regulate the reduction potential of the crystallization solution. While they do provide some hypotheses for why this happened, discussion related to reduction potentials should be included. Additionally, if it is easier to isolate the P1+ state in Mi than in other nitrogenases, this could also be corroborated with spectroscopy.

This is a very fair point, and we acknowledged the reviewer's argument. While EPR spectroscopy experiments are beyond the scope of the present study, we have clarified throughout the text that our observations could be corroborated by future spectroscopic experiments (please see lines 192-193, and 249-250 in the revised manuscript). Instead of using the term P^{3+}, we now refer to the observed state as “the second oxidised state”.
The redox potential of the previously reported P^{1+} is now cited.
Lines 57-58: “Midpoint potentials for P^N/P^{1+} and P^{1+}/P^{2+} couples have been reported to be −310 mV at pH 7.5–8.0 (https://doi.org/10.1021/acs.chemrev.9b00663).”
We will do our best to perform the EPR spectroscopy in future work. However, this task will be very challenging given the low biomass yield obtained from this methanogen when it is grown under N_2-fixation.

(7) Turnover state in the presence of O2 without FeP? -The authors clearly hypothesize that their "form B" or "turnover state" may have arisen from "crystallization delay... uncontrolled oxidation event (e.g., partial O2 contamination in the glove box)..." but then claim that the form B's FeMoco is in a "turnover state". In the discussion, (lines 266-267) they state "...as well as the rapid purification and crystallization under strict exclusion of O2 and dithionite..." as why form B exists. These statements are directly contradictory, and I am not convinced of a "turnover state" when the authors themselves have doubts about the oxygen exclusion of their glove box. I do think the comparison to other turnover states is interesting. It might be in a state resembling an intermediate- I just don't understand how that could happen with (1) oxygen in the glovebox and (2) no reductant of MoFeP (ie no FeP). The authors would need to show experiments demonstrating this ability of the nitrogenase from this organism to reduce substrates (such as N2) in the presence of DTT without FeP for this to be a convincing sound hypothesis.
Form A (containing 100% FeMoco resting state) is the one that has been oxidised. “The crystallisation delay in form A would have provoked a further uncontrolled oxidation event (e.g., partial O_2 contamination in the glove box)”. Form B (harbouring the mixture of the resting and turnover states) appeared very quickly and was immediately harvested. Therefore, the statement was correct, but we rephrased the sentence to clarify the message
Lines 206-212: “Form A comes from a low-concentrated sample with crystals appearing after a month. In contrast, form B was obtained from a high-concentrated sample, and crystals were immediately flash frozen after appearing within a few days. (Fig. 4c). This explanation implies that during harvesting and lysis, the extracted *Mi*NifDK harboured in the majority a P^{1+} state, which was stable enough to be maintained over the purification and fast crystallisation by the intrinsic enzyme rigidity and the mild reductant dithiothreitol. Dithiothreitol did not cause the P-cluster reduction to P^N in contrast to strong reducing agents such as dithionite (Extended Data Fig. 1e)[16]”
Based on our interpretation and previous work on the V- and Fe-only nitrogenases, the so-called “turnover state” should not be due to oxidation, and the bound ligand has not yet been experimentally characterised. The fact that the P-cluster in form B is attributed to P^{1+} based on the electron density also points to an absence of oxidation. We also used a mild reductant (2 mM dithiothreitol) to preserve the protein.

We perfectly understand the argument of the reviewer that the observed state deviating from the resting state could be misinterpreted by calling it "turnover state". However, Einsle and coworkers introduced this nomenclature on the "turnover state" in the V-nitrogenase (Science, 2018) and Fe-only nitrogenase (Nature Catalysis, 2023) in several reports and reviews. Both experimental descriptive works on the turnover state have already been cited 373 times. Therefore, we think that changing this nomenclature while we observe the same state will be confusing for readers and experts in the field. Therefore, we would like to keep this term.

We have now further clarified that the "turnover state" was introduced by Einsle and may not reflect an intermediate stage of the Lowe-Thorneley model.

Lines 242-244: "Whether the conformation truly represents a turnover state remains a matter of debate, and it might not reflect an intermediate stage of the Lowe-Thorneley model. However, because of its usage in literature, we kept this nomenclature in the present study. "

(8) Missing key citations The following pieces of information lack vital citations:

- Line 59 "...Azotobacter vinelandii, whose disproportionately large set of nif genes..." - doi: 10.1128/jb.171.2.1017-1027.1989
- Line 104 "...the maximal temperature allowed by the ATP-regeneration system..."- provide a citation for this (I don't know of one) or provide an SI figure showing data to support this
- Line 119 "... canonical NifD2K2 assembly in which dimerization occurs via NifK..." – cite https://doi.org/10.1021/bi00079a006
- Line 151-152 "... known for its high intrinsic flexibility and participating in FeMoco delivery and catalysis" – should also cite DOI: 10.1126/science.abq7641
- Lines 176-177- "... was proven by exchanging the air of Phe99K/Ser188K to Tyr99K/Ala188K in AvNifDK."- cite https://doi.org/10.1021/jacs.9b04555
- Line 213- "... corroborating that the FeMoco is chemically identical to that of AvNif..." this should read "corroborating that the FeMoco is chemical identical to that of Av NifDK..." and should cite the following 2 papers that identified the central atom of FeMoco to be a carbide: DOI: 10.1126/science.1214025 and DOI: 10.1126/science.1206445

The suggested citations have all been added to the revised manuscript.

(9) Figures: The following figures need modifications:

• About a quarter of the intro focuses on the clusters and ET. The reader must understand this to understand the results and discussions later in the manuscript. I think it would be highly beneficial to move extended data fig 1a to the main text for this reason.

Based on this comment and the one from reviewer 3, we have now moved Extended Data Fig 1 as main Figure 1 and added the P-cluster states reported in the literature.

• Figure 1- the legends in 1a and 1b are swapped. Protein bands in 1d should be labeled similar to 1c. It looks like there is still NifI, even when it is supposed to be removed.

Thanks for pointing that out. We fixed this mistake and completed Fig 1d. The point of $NifI_{1,2}$ has been addressed in a comment above (please see point 2).

• Figure 2- It is very hard to see the "transparent white" portion.
Thanks to the suggestion of reviewer 3, we have now replaced the left panel of old Figure 2c (new Figure 3e) with the overall positions of the hot spots from new Extended Data Fig. 6. The overlay of *Av*NifDK and *Mi*NifDK has now been added to the new Extended Data Fig. 4.

• Figure 3- The transparency/depth cuing on this figure seems off. It is hard to discern what is important and where residues are in relation to the clusters. Also, making the labels of the residues match in transparency to the depiction of them in the model would be more clear than having all labels at 100% opacity. Also, indicating the ET chain would be helpful.
We did our best to improve the new figure 4 according to the referee's suggestions:
1) The depth has been increased
2) All visible residues now have a line pointing to the respective label
3) Cartoons were reduced, and their transparency increased to focus on the residues.
4) A complete turn of 180° is now presented to visualise all residues and simplify the representation.
5) The electron transfer chain is now shown as a dashed line with the written distance.

• Figure 4- I am fairly certain that the models shown are the structures from this paper and not from the PDB, but it is unclear in the caption and main text. "1st and 2nd spheres" should say primary and secondary coordination spheres. I also think calling the structure a turnover structure could be misleading.
New Figure 5 and its legend have been updated to clarify that the models shown originate from previous work. The structures from the PDB used for sequence and structural comparisons in Figs. 3 and 5, and for the Extended Data Figs. 2, 3, 4, and 8 are specified in lines 509-510 of the revised manuscript. Please see our previous point regarding nomenclature on the resting and turnover states introduced by Einsle and colleagues.

• Extended figure 1b- the colors in the plot don't match the legend. Added N2 in mmol isn't a very useful measure. Could this be converted to concentration (partial pressures)? Also, it should be mentioned in the caption that "as isolated" is DTT reduced.
We changed the colour in the new Fig 1b for NifD-like. If the reviewer meant Extended Data Figure 2b, then the legends and the plot fit the colour coding, and we cannot find any discrepancies.
Based on Reviewer 3's comments, we homogenised all units in the figures. Added NH_4^+ is now in mmol, a unit that is indeed important for understanding the relationship between the different experiments.

(9) Other comments that need to be addressed:
• When the amount of N2 used during growths is mentioned throughout the paper (main text and figs), it says the amount in mmol. Putting this in terms of concentration such as partial pressures makes more sense.

As described in the comment above, this point has been addressed in the revised manuscript.

• Line 110 could be further clarified by adding to the end: "... stalling the enzyme at lower temperatures.

The sentence referenced above was clarified as suggested (please see the line 118 in the revised manuscript).

• The central atom in the P-cluster is called a "sulfur" throughout the manuscript, but it should be "sulfide".

The main text was corrected as suggested (please see the lines 177, 179, 182, 195, 199 and 242 in the revised manuscript).

• For the paragraph beginning at line 164, it would be beneficial to add an intro sentence or two that clearly and concisely introduces the known oxidation states of the P-cluster and their corresponding conformational changes, with appropriate citations. As it is currently written, it is scattered throughout the paragraph and isn't clear to readers who might not be intimately familiar with the P-cluster. Rather than citing a review, cite https://doi.org/10.1021/bi9626665 and https://doi.org/10.1074/jbc.RA118.002435 (citation #57 in this manuscript)

The description of the known P cluster oxidation states has now been incorporated into the new Figure 1, and the introduction, and the suggested references have been included (please see the lines 57-61 in the revised manuscript).

• Line 165 should be clarified with "This 2e– oxidized state..."

The sentence referenced above was clarified as suggested (please see line 175 in the revised manuscript).

• The authors say that they don't use diothionite during purification and crystallization because it could possibly be a "non-innocent reductant for metalloproteins". However, they still use it in their activity assays as the sacrificial electron donor. To me, their reason for not using it thus isn't justified, and they need to clarify in the main text that they still used it in the assays, and an explanation as to why it is ok to use in assays but not other parts of their methods (which I don't understand).

In the activity assays, under high turnover conditions, the role of dithionite is transient. We use it as an electron donor to reduce the Fe-protein (NifH) for up to 30 minutes. However, adding dithionite throughout the entire procedure (cell harvesting, cell lysis, purification, crystallisation) will result in the accumulation of undesired side products in contact with the nitrogenase over days, which could bias the structural characterisation presented in this work. Therefore, we refrain from adding dithionite everywhere, since it has been reported to generate a myriad of undesirable side products over time (Telfeyan K. et al., Applied Geochemistry, 2019), and could interfere with metallo-containing enzymes (e.g., Martini MA et al., JACS, 2021).

• In the author's previous paper (Maslać et al. 2022 Comparative Transcriptomics Sheds Light on Remodeling of Gene Expression during Diazotrophy in the Thermophilic Methanogen

Methanothermococcus thermolithotrophicus), the authors state "a supplementation with the concentration of 100 uM [W] gave unreproducible results". In this manuscript, the authors use 100uM, citing the aforementioned paper. Can the authors please explain why they changed growth media to one that previously gave unreproducible results?

In our previous paper (Maslać et al. 2022 Comparative Transcriptomics Sheds Light on Remodeling of Gene Expression during Diazotrophy in the Thermophilic Methanogen *Methanothermococcus thermolithotrophicus*), a different model organism was used: namely, the marine thermophile *Methanothermococcus thermolithotrophicus*. In the present manuscript, the medium was adapted to the model used here, the hyperthermophile *Methanocaldococcus infernus*, which grows reproducibly during diazotrophy in the medium containing 100 µM [WO_4^{2-}].

Reviewer #3:

(1) Line 38: How about providing a brief overview of the redox states of the P-clusters and the FeMoco site of nitrogenase, the two main observations in this report?

The description/overview of both nitrogenase cofactors (P-cluster and FeMoco) oxidation states has now been incorporated into the introduction (please see the lines 57-63 in the revised manuscript) and in the new Figure 1.

(2) Line 51-55: For the general readers, it would be helpful to include a more detailed structural description of the P-cluster, FeMoco, FeV, and FeFe cofactors, including their compositions such as MonFenSn..., in the main text.

The structural description and chemical composition of the nitrogenase cofactors (P cluster and FeMo-co as well as its isoforms) have now been incorporated into the introduction (please see the lines 51-55 and 63 in the revised manuscript).

(3) Line 76: It seems that Figures 1a and 1b have been mixed up. Please check the figures.

Thanks for pointing that out. We fixed this mistake.

(4) Line 79: Why is mol mass (mmol) used in the text rather than concentration (mM) used in the figures? For easy comparison, please use only one unit. For example, change the unit in the figures to molar mass.

The units for NH_4Cl in Figure 1 have now been changed to mmol, which is being used uniformly throughout the revised manuscript.

(5) Line 125: If possible, provide an X-ray fluorescence edge scan of the Mo and Fe, and indicate the energy at which anomalous data were collected in an extended data figure.

We agree that an X-ray fluorescence (XRF) scan can, in principle, provide useful information on the elemental composition of a crystal. We therefore attempted to measure an XRF spectrum on the same crystal that was subsequently used for diffraction data collection above and below the Mo K-edge.

For this tentative XRF measurement, the incident X-ray energy was set to 20.1 keV, approximately 100 eV above the theoretical Mo K-edge. The measurement was performed using a KETEK fluorescence detector, with a 300 × 300 µm beam size and 50% transmission.
The resulting spectrum indicated (see figure below) the presence of potassium and/or chloride, as well as iron. However, under the available experimental conditions, the identification of molybdenum in the XRF spectrum was inconclusive. In particular, 20.1 keV was the highest incident X-ray energy accessible on the beamline because of mechanical constraints associated with the optics. At this energy, the incident beam strongly affected the fluorescence signal in the Mo K-emission region, which prevented an unambiguous assignment of the Mo fluorescence lines.

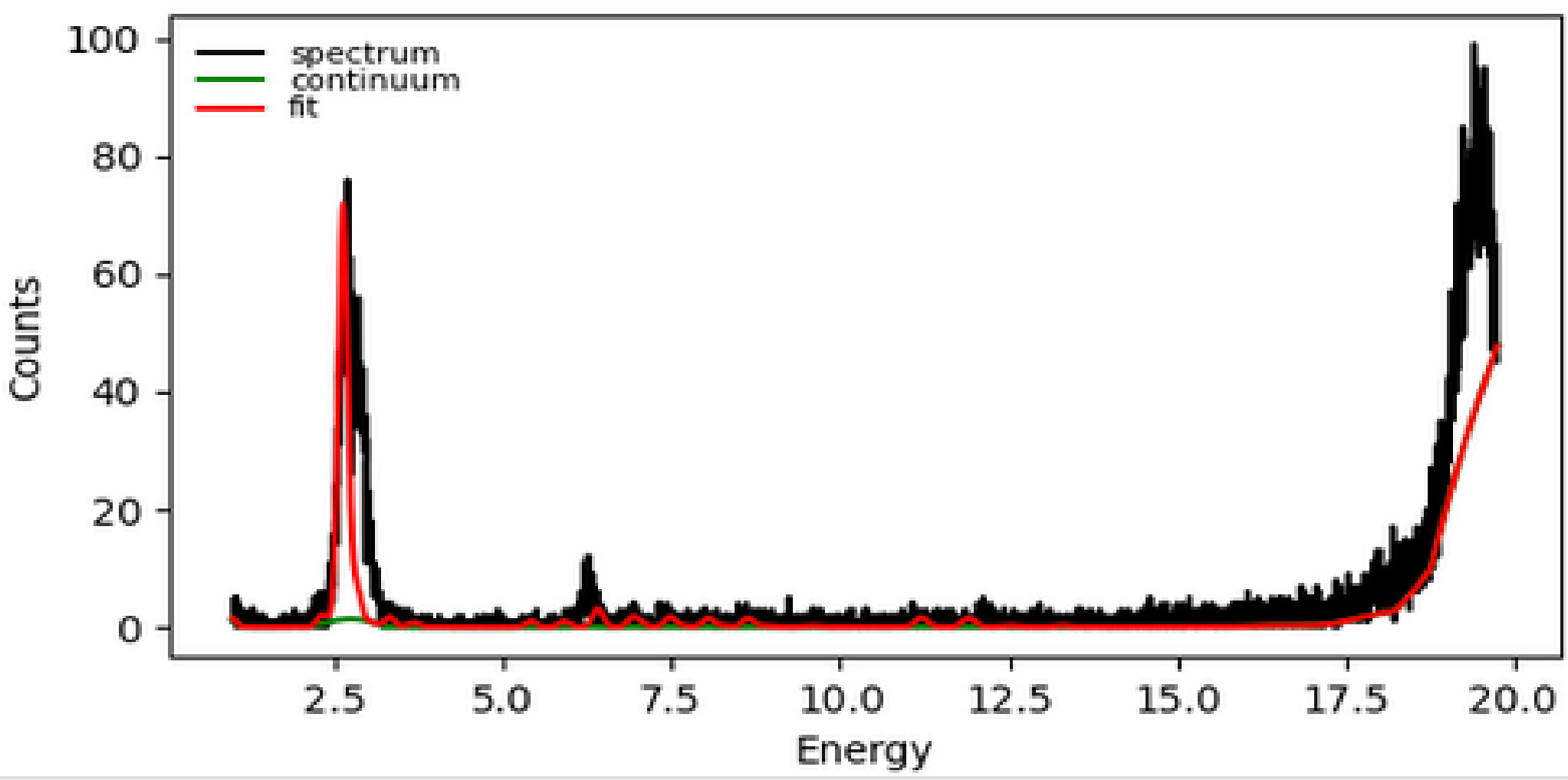

Figure. XRF spectrum recorded on BM07-FIP2 beamline at ESRF

Nevertheless, we would like to emphasise that the approach used in the manuscript provides substantially stronger evidence for the presence of molybdenum in the active site than a standalone XRF spectrum. Indeed, XRF would only confirm the presence of molybdenum somewhere within the irradiated crystal volume, or potentially in the surrounding mother liquor. By contrast, our diffraction experiment was specifically designed to probe the anomalous contribution of molybdenum by collecting data above and below the Mo K-edge. The resulting anomalous difference maps allow us to spatially localise the anomalous peak from the Mo within the protein structure.
Thus, although the XRF spectrum could not provide an unambiguous molybdenum assignment under the available beamline conditions, the above/below-edge diffraction strategy unambiguously identifies molybdenum at the active site and provides more direct structural evidence for its presence than XRF alone.

The energy at which anomalous data have been collected is now specified in the main text (please see lines 133-136 in the revised manuscript).

(6) Line 161: Define the superscripts K and D when they are first used in the text.

The superscripts K and D used to indicate residues belonging to Nif/Vnf/AnfK and Nif/Vnf/AnfD have been clarified in the main text (please see lines 171-172 in the revised manuscript).

(7) Line 181: Clearly describe conformations 1 and 2 of the P1+ state in the text. Change 'the P1+ state in which' to 'the P1+ state (70% occupancy of conformation 1) in which'.
The text was modified as suggested (please see lines 194-195 in the revised manuscript).

(8) Line 184: Change 'occupancy of 30%' to 'occupancy of 30% (Conformation 2)'.
The text was modified as suggested (please see line 198 in the revised manuscript).

(9) Line 185: Indicate new bonds for the P1+-conformation 2 (Fe5 to the amide backbone of Cys72D and Fe6 to the central S) by arrow or star on the fig. b, as these are difficult to identify at first glance.
New Figure 4b was modified accordingly.

(10) Line 210: Indicate the H-bonding network in Fig. 4a, for example using a dashed bond.
New Figure 5a was modified accordingly to reflect the presented H-bonding network between homocitrate and residues. For clarity reasons, the H-bonding network of the residues themselves is not displayed

(11) Line 479-486: Please incorporate this paragraph into the legend of Fig. 2a or the main text where appropriate, as it is not very convincing and it is difficult to understand the type of maps presented and how they are made. If this is not feasible, please refer to the "Methods" section for a detailed explanation of how the maps are calculated and what they are.
The cited paragraph is now incorporated into the legend of new Figure 3a, along with the reference to the Methods (please see lines 585-589 in the revised manuscript), and clarified in the main text as well (please see lines 133-136 in the revised manuscript).

(12) Line 481: change "A difference maps" to A anomalous difference Fourrier maps".
The text was modified as suggested (please see line 502 in the revised manuscript).

(13) Line 529: In Fig. 1b, comment on why the culture exhibits a longer lag phase in the presence of 100 μM Na_2MoO_4.
While we cannot conclusively claim this is the case here, there is evidence that increased trace metal concentrations in general can lead to an increase in the duration of the lag phase, as reported in some other works with methanogens (e.g. https://www.sciencedirect.com/science/article/pii/S2213343723002671#sec0005). We suspect that such a high concentration of molybdate cross-reacts with other compounds in the medium, potentially poisoning the methanogen.

(14) Line 543:

1) Change 'Anomalous difference maps' to 'Anomalous difference Fourier maps'.

The text was modified as suggested (please see line 584 in the revised manuscript).

2) Are these maps calculated from the high-energy side, or are the double difference maps calculated from the high-energy side minus the low-energy side? If so, please mention in the legend text how the map was calculated from the low-energy side.

These maps have been calculated from the high-energy side minus the low-energy side, and the legend is now complete.

3) In the figure legend, mention the energy at which the anomalous difference Fourier maps were calculated on the high-energy side (xx Å) or the low-energy side (yy Å) of the metal absorption edge.

Legend has been completed as suggested.

(15) Line 545: in the right graph of Fig. 2a, what does 'overall' mean? It seems that the authors indicated it as 'NifD2K2'. If this is the case, change 'overall' to 'NifD2K2'.

We removed the overall and rephrased the legend to avoid any ambiguity

Lines 593-594: "c, Comparison of the surface accessibility and molecular weight between *Mi*NifDK overall structure and structural homologues".

(16) Line 548: in the left panel of fig. 2c,

1) There are 11 red spheres representing the hot spots, but only 10 are shown in the extended data Fig. 7. What about the 11th sphere? Would it be from other subunit. Please comment on this.

   One of the additional red spheres was localised near another and was merged into a single hot spot in the previous Extended Data Fig. 7. We followed the reviewer's suggestion to integrate the sub-panel of the new Extended Data Fig. 6 into new Fig. 3e. Now, 10 hot spots are presented in all figures in the revised version.

2) The reviewer does not see the benefit of superposing MiNifDK and AvNifDK, or of showing the absence of metal clusters, in the figure. Since the comparison of the two structures for the hot spots is clearly shown in the extended data Fig. 7, it is not necessary to show the superposition again here, as this actually makes the view more complex. The reviewer thinks that the structure in the top right panel of the extended data Fig. 7 is more informative and would fit better in the main figure.

   This is an excellent point that will simplify the information overload in the previous Figure 2c. Accordingly, we replaced the panel in the previous Extended Data Figure 7 with a novel panel in Fig. 3 and moved the *Mi*NifDK/*Av*NifDK superposition to the new Extended Data Figure 4.

(17) Line 550: in the fig. 2c right graphs for the number of hydrogen bonds/salt bridges, what stands for dark and light green bars for?

The dark and light green bars correspond to the intramolecular hydrogen bonds and intramolecular salt bridges, respectively. This is now clarified in the new Figure 3d on the Y-axis.

(18) Line 572: In Fig. 4a, 'Q462K′' is written, but there is no explanation of the " ′ " in the legend.

The superscripts " ′ " used to differentiate between the two different subunits of NifK and NifD have been clarified in the legend of Figure 4 (please see lines 588-589 in the revised manuscript).

(19) Line 583: In Extended Data Table 1,

1) the Form A crystal shows an increase in ⟨I/σ(I)⟩ with increasing X-ray energy, as expected. However, for the two anomalous Mo data sets collected at 19.9 and 20.1 keV, ⟨I/σ(I)⟩ decreases as the data-collection energy increases. In addition, higher-energy data collection is generally expected to allow access to higher-resolution diffraction, yet this is not observed for the Form B crystal.

We agree that, in the absence of other effects, increasing the X-ray energy can reduce absorption and may improve the diffraction statistics and/or the accessible resolution. This trend is indeed observed for the Form A crystal. However, we do not find the decrease in I/sig(I) between the two Mo anomalous data sets collected at 19.9 and 20.1 keV unexpected. These two energies lie on opposite sides of the Mo K absorption edge, and the data set collected above the edge is therefore affected by the abrupt increase in absorption by the Mo-containing sample. The stronger absorption above the edge reduces the diffracted intensities and can consequently lead to poorer statistics, despite the slightly higher incident photon energy. Similar behaviour has been observed previously for anomalous data collection across an absorption edge, for example in the Kr-edge experiments reported by Engilberge et al. Chem. Comm. 2020. In addition, the PILATUS3 6M detector used for these measurements is based on a silicon sensor. At energies around 20 keV, the quantum efficiency of the sensor is already reduced (table below).

Quantum efficiency of Si Pilatus 6M from media.dectris.com « technical specification »

Table 3.1: Quantum Efficiency

Sensor thickness[1]		450 µm	1000 µm
Quantum efficiency at	5.4 keV (Cr)	94 %	> 80 %
	8.0 keV (Cu)	98 %	96 %
	17.5 keV (Mo)	47 %	76 %

Although this does not account for the observed change, it means that both measurements were performed in a relatively low-efficiency regime of the detector, making the statistics more sensitive to additional absorption losses in the sample.

Finally, the absence of improved high-resolution diffraction for the Form B crystal at higher energy is not necessarily contradictory. The maximum observed resolution is not determined only by the X-ray energy, but also by crystal quality, crystal volume and more importantly in this case, beamline flux and exposure conditions. To reach the Mo edge, we pushed the beamline optics to their limit. In particular, the mirrors were set at a very grazing angle, which strongly reduced the flux at the sample position. This contributed to a lower high-resolution signal. However, we consider this acceptable because the data were collected primarily to

compute an anomalous Fourier map, for which the available resolution and anomalous signal were sufficient.

2) The number of unique reflections can be approximately doubled when anomalous data are processed with Friedel mates treated as independent reflections, rather than being merged. However, for the datasets presented here, the number of reflections between the anomalous data and the high-resolution data does not match this expectation.

Please comment on these observations. Additionally, indicate each data set how you treated the Friedel mates for data processing.

We agree that, for a given crystal form and resolution limit, treating Friedel mates as independent observations in anomalous data processing can approximately double the number of unique reflections compared with merged non-anomalous data. However, this comparison only holds when the datasets are considered to be at the same high-resolution cut-off. In the present case, the anomalous and high-resolution datasets were processed to significantly different resolution limits. Relatively small changes in the high-resolution cut-off can substantially affect the total number of unique reflections. Therefore, the number of unique reflections in the anomalous datasets is not expected to be simply twice that of the corresponding high-resolution datasets.

(20) Line 620: provide a concentration of the as-isolated MiNifDK in the legend for comparison with the added DT.

The concentration of as-isolated *Mi*NifDK is now provided in the legend of the new Extended Data Figure 2.

(21) Line 689: in Extended data fig. 9a,

1) All the residues indicated are from the NifD subunit (D superscript), particularly Q462D. However, Q462 is from the NifK subunit in Fig. 4a. Check for inconsistencies and correct them.

Thanks a lot for noticing this mistake, which arose from not colouring the chain differently. We corrected the inconsistencies, colored the two chains appropriately, and cross-checked all residues and subunits from the different figures.

We checked twice all the labelling of the figures.

2) There are two asterisks ('*') in H430D/H442D and a His residue in both figures of Fig. 9a. However, there is no explanation in the legend about the meaning of these.

The asterisk refers to the position. We now simply add the label directly close to the residue and remove the asterisks.

Response to the reviewer.

We would like to thank all reviewers for their dedicated attention to our work.

Please find below the answer to the pending remarks of Reviewer 2.

Thank you to the authors for carefully considering the comments during their revision. The revised manuscript by Maslać et al. has made many of the requested revisions. I have just a few minor questions and requested revisions remaining before recommending the manuscript for publication.

Omit maps of the P-clusters – I find the identification of the percent occupancy of different states of the P-clusters in the crystals very interesting. This would be even more convincing with proper omit maps as extended data figures, which I would expect to show elongation/multiple positions around relevant atoms/residues.

Based on the reviewer´s comment, we have now removed the omit map from the new Extended Data Fig. 7 and created a new supplementary figure (Supplementary Figure 1) with omit maps for all states of the P-clusters reported in this study.

1. **Thank you for including the omit maps. Can you please label the metal centers and include the omitted ligands of interest (C72 and S143) to make the figures less ambiguous?**

(Our response) We have now updated the Supplementary Figure 8 by adding the ligands of interest, C72 and S143 and the bridging water molecule. Omit maps for the ligands are now presented as additional panels in Supplementary Figure 8.

2. **There are a few new sentences in the manuscript introduction that could use some editing (lines 52-63).**

Original: *FeMoco is asymmetric, adopting a trigonal prismatic (D3h) geometry: with two prism halves linked by three bridging sulfides, contains central μ_6-carbide14,15, and an apical molybdenum atom. The P-cluster, harboured at the NifDK interface, consists of two symmetric [4Fe:4S] cubanes that share a central μ6-sulfur (S) atom in its reduced state. During catalysis, the P-cluster has been proposed to oscillate between a fully reduced PN and the electron-depleted P1+ state (following the accepted deficit-spending mechanism, Fig. 1a)16-18. Midpoint potentials for PN/P1+ and P1+/P2+ couples have been reported to be −310 mV at pH 7.5–8.016. While the P-cluster can be further oxidised to P2+ and P3+ states19,20, there is no evidence that these transitions happen during the catalytic cycle15,19. Moreover, the P3+ state is considered irreversible21. The FeMoco can also oscillate between two different states during catalytic turnover: stable resting S=3/2 state (FeMocoN) and one-electron oxidised state (FeMocoox)16. There are two more isoforms of nitrogenase in which the FeMoco is*

replaced by a FeV-cofactor (Vnf system, [V:7Fe:8S:C:CO32-]) or a FeFe-cofactor (Anf system, [8Fe:9S:C], Fig. 1a)22,23.

Suggestion: *FeMoco is asymmetric, adopting a trigonal prismatic (D3h) geometry [remove colon] with two prism halves linked by three bridging sulfides, containing a central μ_6-carbide14,15 [remove comma] and an apical molybdenum atom. The P-cluster, harboured at the NifDK interface, consists of two symmetric [4Fe:4S] cubanes that share a central μ6-sulfide (S) in its reduced state. During catalysis, the P-cluster has been proposed to oscillate between a fully reduced PN and the electron-depleted P1+ state (following the accepted deficit-spending mechanism, Fig. 1a)16-18. Midpoint potentials for the PN/P1+ and P1+/P2+ couples have been reported to be −310 mV at pH 7.5–8.016. While the P-cluster can be further oxidised to P2+ and P3+ states19,20, there is no evidence that these transitions happen during the catalytic cycle15,19. Moreover, the P3+ state is considered irreversible21. [Remove "The"]FeMoco can also oscillate between multiple different states during catalytic turnover including the stable S=3/2 resting state (FeMocoN) and the one-electron oxidised state (FeMocoox)16. There are two more isoforms of nitrogenase in which [remove "the"] FeMoco is replaced by a FeV-cofactor (Vnf system, [V:7Fe:8S:C:CO32-]) or a FeFe-cofactor (Anf system, [8Fe:9S:C], Fig. 1a)22,23.*

We thank the reviewer for the text improvement suggestions: the text was revised as suggested above.

3. **Regarding both of the following comments:**

Extended figure 1b- the colors in the plot don't match the legend. Added N2 in mmol isn't a very useful measure. Could this be converted to concentration (partial pressures)? Also, it should be mentioned in the caption that "as isolated" is DTT reduced.

We changed the colour in the new Fig 1b for NifD-like. If the reviewer meant Extended Data Figure 2b, then the legends and the plot fit the colour coding, and we cannot find any discrepancies. Based on Reviewer 3's comments, we homogenised all units in the figures. Added NH4+ is now in mmol, a unit that is indeed important for understanding the relationship between the different experiments.

When the amount of N2 used during growths is mentioned throughout the paper (main text and figs), it says the amount in mmol. Putting this in terms of concentration such as partial pressures makes more sense.

I am still confused why the additives are not in concentration/partial pressures. This makes it difficult to compare reaction conditions because the reader must go though the methods to find the total culture volumes to know the concentration. Adding 0.22 mmol of ammonium to 10 mL of culture is very different than adding that same amount to 10 L of culture, for example.

We apologise for this oversight and have added the partial pressure to Supplementary Figure 1b and to the legend of Figure 2 panels a and b. The partial pressure calculations are now stated in the method section. The relevant text was accordingly updated.

Thank you to the authors for carefully considering the comments during their revision. The revised manuscript by Maslać et al. has made many of the requested revisions. I have just a few minor questions and requested revisions remaining before recommending the manuscript for publication.

- Omit maps of the P-clusters – I find the identification of the percent occupancy of different states of the P-clusters in the crystals very interesting. This would be even more convincing with proper omit maps as extended data figures, which I would expect to show elongation/multiple positions around relevant atoms/residues.
  Based on the reviewer´s comment, we have now removed the omit map from the new Extended Data Fig. 7 and created a new supplementary figure (Supplementary Figure 1) with omit maps for all states of the P-clusters reported in this study.
  Thank you for including the omit maps. Can you please label the metal centers and include the omitted ligands of interest (C72 and S143) to make the figures less ambiguous?
- There are a few new sentences in the manuscript introduction that could use some editing (lines 52-63).

  Original: FeMoco is asymmetric, adopting a trigonal prismatic (D3h) geometry: with two prism halves linked by three bridging sulfides, contains central μ_6-carbide14,15, and an apical molybdenum atom. The P-cluster, harboured at the NifDK interface, consists of two symmetric [4Fe:4S] cubanes that share a central µ6-sulfur (S) atom in its reduced state. During catalysis, the P-cluster has been proposed to oscillate between a fully reduced PN and the electron-depleted P1+ state (following the accepted deficit-spending mechanism, Fig. 1a)16-18. Midpoint potentials for PN/P1+ and P1+/P2+ couples have been reported to be −310 mV at pH 7.5–8.016. While the P-cluster can be further oxidised to P2+ and P3+ states19,20, there is no evidence that these transitions happen during the catalytic cycle15,19. Moreover, the P3+ state is considered irreversible21. The FeMoco can also oscillate between two different states during catalytic turnover: stable resting S=3/2 state (FeMocoN) and one-electron oxidised state (FeMocoox)16. There are two more isoforms of nitrogenase in which the FeMoco is replaced by a FeV-cofactor (Vnf system, [V:7Fe:8S:C:CO32-]) or a FeFe-cofactor (Anf system, [8Fe:9S:C], Fig. 1a)22,23.

  Suggestion: FeMoco is asymmetric, adopting a trigonal prismatic (D3h) geometry [remove colon] with two prism halves linked by three bridging sulfides, containing a central μ_6-carbide14,15 [remove comma] and an apical molybdenum atom. The P-cluster, harboured at the NifDK interface, consists of two symmetric [4Fe:4S] cubanes that share a central µ6-sulfide (S) in its reduced state. During catalysis, the P-cluster has been proposed to oscillate between a fully reduced PN and the electron-depleted P1+ state (following the accepted deficit-spending mechanism, Fig. 1a)16-18. Midpoint potentials for the PN/P1+ and P1+/P2+ couples have been reported to be −310 mV at pH 7.5–8.016. While the P-cluster can be further oxidised to P2+ and P3+ states19,20, there is no evidence that these transitions happen during the catalytic cycle15,19. Moreover, the P3+ state is considered irreversible21. [Remove "The"]FeMoco can also oscillate between multiple different states during catalytic turnover including the stable S=3/2 resting state (FeMocoN) and the one-electron oxidised state (FeMocoox)16. There are two more isoforms of nitrogenase in which [remove "the"] FeMoco is replaced by a FeV-cofactor (Vnf system, [V:7Fe:8S:C:CO32-]) or a FeFe-cofactor (Anf system, [8Fe:9S:C], Fig. 1a)22,23.

Regarding both of the following comments:

- Extended figure 1b- the colors in the plot don't match the legend. Added N2 in mmol isn't a very useful measure. Could this be converted to concentration (partial pressures)? Also, it should be mentioned in the caption that "as isolated" is DTT reduced.
  We changed the colour in the new Fig 1b for NifD-like. If the reviewer meant Extended Data Figure 2b, then the legends and the plot fit the colour coding, and we cannot find any discrepancies. Based on Reviewer 3's comments, we homogenised all units in the figures. Added NH4+ is now in mmol, a unit that is indeed important for understanding the relationship between the different experiments.
- When the amount of N2 used during growths is mentioned throughout the paper (main text and figs), it says the amount in mmol. Putting this in terms of concentration such as partial pressures makes more sense.
  As described in the comment above, this point has been addressed in the revised manuscript.
- I am still confused why the additives are not in concentration/partial pressures. This makes it difficult to compare reaction conditions because the reader must go though the methods to find the total culture volumes to know the concentration. Adding 0.22 mmol of ammonium to 10 mL of culture is very different than adding that same amount to 10 L of culture, for example.
